# Vaginal and rectal microbiome contribute to genital inflammation in chronic pelvic pain

**DOI:** 10.1186/s12916-024-03500-1

**Published:** 2024-07-08

**Authors:** Nicole Jimenez, Taylor Norton, Gurbeen Diadala, Emerald Bell, Michelle Valenti, Leslie V. Farland, Nichole Mahnert, Melissa M. Herbst-Kralovetz

**Affiliations:** 1 Department of Obstetrics and Gynecology, College of Medicine-Phoenix, University of Arizona, Phoenix, AZ USA; 2 Department of Obstetrics and Gynecology, Banner University Medical Center Phoenix, Phoenix, AZ USA; 3 Basic Medical Sciences, College of Medicine-Phoenix, University of Arizona, Building ABC-1, Lab 331E, 425 N. 5 St, Phoenix, AZ 85004 USA; 4 UA Cancer Center, University of Arizona, Tucson, AZ USA; 5 Department of Epidemiology and Biostatistics, Mel and Enid Zuckerman College of Public Health, University of Arizona, Tucson, AZ USA; 6 Department of Obstetrics and Gynecology, College of Medicine Tucson, University of Arizona, Tucson, AZ USA; 7 University of Arizona College of Nursing, Tucson, AZ USA

**Keywords:** Chronic pelvic pain, Endometriosis, Abnormal uterine bleeding, Fibroids, Vaginal microbiome, Gut microbiome, Women’s health

## Abstract

**Background:**

Chronic pelvic pain (CPP) is a multifactorial syndrome that can substantially affect a patient’s quality of life. Endometriosis is one cause of CPP, and alterations of the immune and microbiome profiles have been observed in patients with endometriosis. The objective of this pilot study was to investigate differences in the vaginal and gastrointestinal microbiomes and cervicovaginal immune microenvironment in patients with CPP and endometriosis diagnosis compared to those with CPP without endometriosis and no CPP.

**Methods:**

Vaginal swabs, rectal swabs, and cervicovaginal lavages (CVL) were collected among individuals undergoing gynecologic laparoscopy. Participants were grouped based on patients seeking care for chronic pain and/or pathology results: CPP and endometriosis (CPP-Endo) (*n* = 35), CPP without endometriosis (*n* = 23), or patients without CPP or endometriosis (controls) (*n* = 15). Sensitivity analyses were performed on CPP with endometriosis location, stage, and co-occurring gynecologic conditions (abnormal uterine bleeding, fibroids). 16S rRNA sequencing was performed to profile the microbiome, and a panel of soluble immune mediators was quantified using a multiplex assay. Statistical analysis was conducted with SAS, R, MicrobiomeAnalyst, MetaboAnalyst, and QIIME 2.

**Results:**

Significant differences were observed between participants with CPP alone, CPP-Endo, and surgical controls for body mass index, ethnicity, diagnosis of ovarian cysts, and diagnosis of fibroids. In rectal microbiome analysis, both CPP alone and CPP-Endo exhibited lower alpha diversity than controls, and both CPP groups revealed enrichment of irritable bowel syndrome-associated bacteria. CPP-Endo exhibited an increased abundance of vaginal *Streptococcus anginosus* and rectal *Ruminococcus*. Patients with CPP and endometrioma (s) demonstrated increased vaginal *Streptococcus*, *Lactobacillus,* and *Prevotella* compared to other endometriosis sites. Further, abnormal uterine bleeding was associated with an increased abundance of bacterial vaginosis-associated bacteria. Immunoproteomic profiles were distinctly clustered by CPP alone and CPP-Endo compared to controls. CPP-Endo was enriched in TNF⍺, MDC, and IL-1⍺.

**Conclusions:**

Vaginal and rectal microbiomes were observed to differ between patients with CPP alone and CPP with endometriosis, which may be useful in personalized treatment for individuals with CPP and endometriosis from those with other causes of CPP. Further investigation is warranted in patients with additional co-occurring conditions, such as AUB/fibroids, which add additional complexity to these conditions and reveal the enrichment of distinct pathogenic bacteria in both mucosal sites. This study provides foundational microbiome-immunoproteomic knowledge related to chronic pelvic pain, endometriosis, and co-occurring gynecologic conditions that can help improve the treatment of patients seeking care for pain.

**Supplementary Information:**

The online version contains supplementary material available at 10.1186/s12916-024-03500-1.

## Background

Chronic pelvic pain (CPP) is a common clinical complaint defined by the pain of the pelvis experienced for greater than or equal to 6 months that substantially burdens a patient’s overall quality of life by affecting physical, emotional, and sexual health [1]. The etiology of CPP can be multifactorial, involving multiple organ systems, making it difficult to diagnose and treat. A common cause of CPP is endometriosis. However, not all women with endometriosis have CPP. Endometriosis is a chronic inflammatory condition defined by endometrium-like tissue outside the uterine cavity [2].

Endometriosis is clinically suspected based on symptoms, physical exam findings, and imaging studies [2]. However, there are currently no validated, non-invasive diagnostic tests for the disease, and definitive diagnosis relies on surgical excision of suspected lesions with histopathology confirmation [3–5]. Frequently, there is considerable delay in endometriosis diagnosis after the onset of pain, with symptomatic patients undergoing evaluation by multiple physicians for 7–10 years before proper diagnosis and treatment [6, 7]. Accurate, non-invasive diagnosis and treatments for CPP with and without endometriosis is a major challenge in the field.

Decades of research have tried to identify diagnostic and therapeutic targets for endometriosis focused on inflammatory and immune markers in the serum or peritoneal and cervicovaginal fluid [4, 8–11]. Unfortunately, no single biomarker or panel has yet been approved for diagnosis in the USA. However, one novel area of investigation that may offer potential targets for diagnosing and treating CPP with and without endometriosis is the microbiome. The female reproductive tract and gut microbiome have become an area of interest related to benign and malignant gynecologic disease [12–20]. Previous research has observed that the microbiome is associated with gynecologic conditions, including bacterial vaginosis [21], yeast infections [22], polycystic ovary syndrome [23, 24] and irritable bowel syndrome [25, 26]. Emerging research into the relationship between the microbiome and endometriosis suggests alterations in the microbiome of the lower and upper reproductive tract, the gastrointestinal system, and within the peritoneal cavity [15, 27–29]. Microbial associations with endometriosis may drive pathogenesis in chronic pelvic pain and endometriosis-associated pain by inducing inflammation [30–32]. However, prior research is limited in investigations of microbial relationships related to CPP alone and how that may differ from patients with CPP and endometriosis [27]. Furthermore, investigating these interactions can lead to therapeutic targets for symptom management.

Given the complex nature of chronic pelvic pain and the established association between endometriosis and alterations in the microbiome, the microbiome could contribute to the pathophysiologic mechanism of pain, specifically chronic pelvic pain outside of endometriosis diagnosis. Further delineation of the relationship between the microbiome, inflammation, endometriosis, and CPP could provide methods for improved treatment to differentiate patients with endometriosis from those with other etiologies of CPP. We hypothesized that the rectal and vaginal microbiome composition and immune profiles would differ between patients seeking care for CPP and endometriosis, CPP without endometriosis, and patients without CPP or endometriosis. These pilot findings yield insight into key microbiome and immunoproteome differences between endometriosis associated CPP and other forms of CPP. Thus, springboarding further investigation in larger cohorts for therapeutic intervention targeting additional scientific questions regarding types of pain, the severity of pain, and additional conditions that co-occur with endometriosis, such as adenomyosis.

## Methods

### Patient recruitment

To investigate the microbiome and local immune microenvironment in chronic pelvic pain and differences among patients with and without endometriosis, we designed a pilot study of gynecologic patients undergoing laparoscopic surgery at a single tertiary academic hospital. After institutional review board approval (#1712142464), patients were recruited and enrolled in a gynecologic surgery practice from 02/07/2019 to 11/29/2021. Surgery types performed included laparoscopic bilateral salpingectomy, total laparoscopic hysterectomy, bilateral salpingectomy, myomectomy, laparoscopic excision of endometriosis, laparoscopic ovarian cystectomy, diagnostic laparoscopy, and laparoscopic oophorectomy. Inclusion criteria included English-speaking, adult (age 19–59) females undergoing gynecologic laparoscopy. Patients were excluded for current pregnancy and current reported infection or antibiotic use for 2 weeks prior or vaginal douching within 24 h.

### Definition of chronic pelvic pain with and without endometriosis

**Fig. 1:**
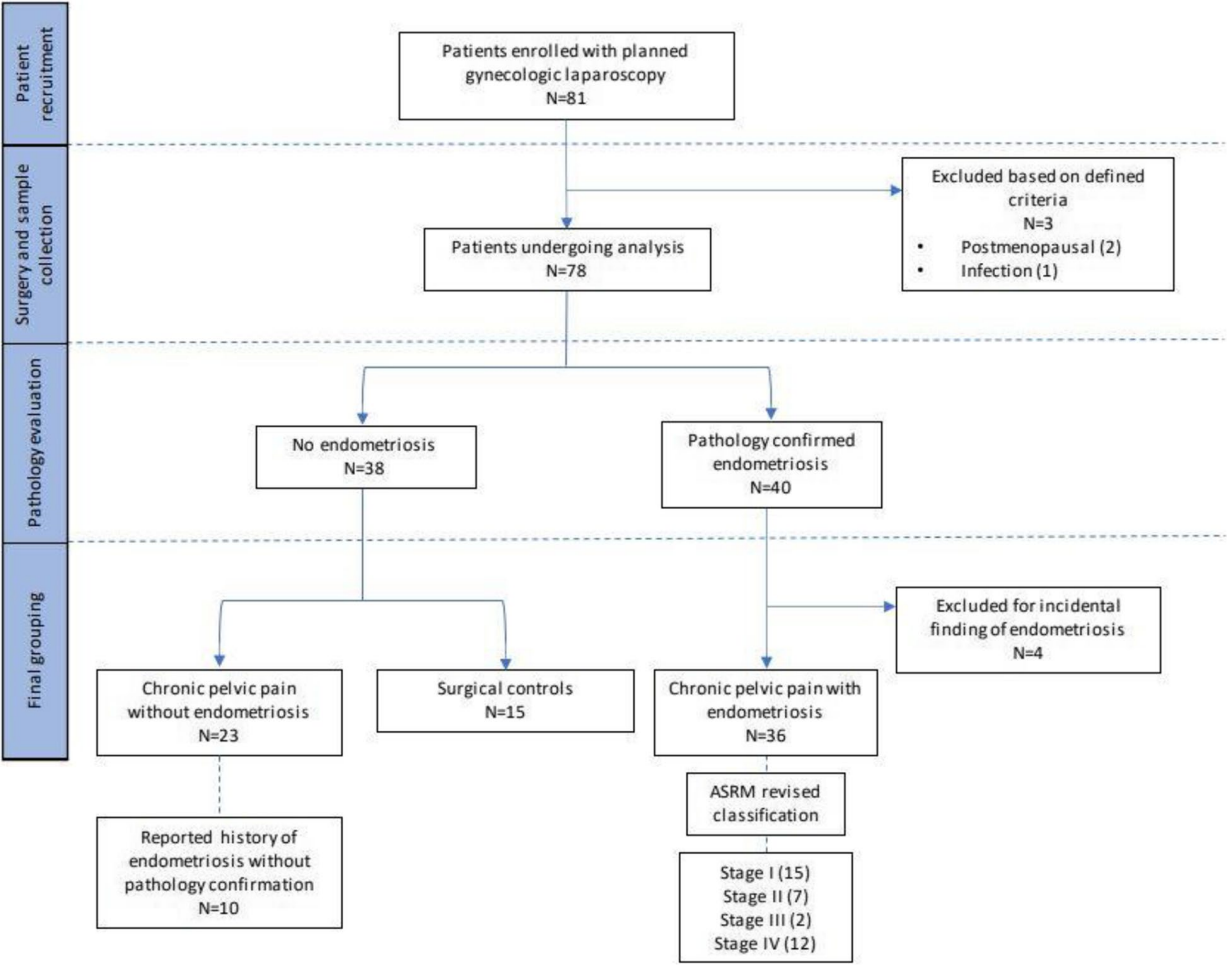
Diagram of cohort groupings and inclusions and exclusion. *N* = 81 women enrolled in the chronic pelvic pain study. Three women were excluded due to exclusion criteria of postmenopausal status or current sexually transmitted infection. Pathology evaluation confirmed diagnosis of endometriosis (*n* = 40) or no endometriosis (*n* = 38). With histological confirmation and symptom diagnosis, we had the following groups: chronic pelvic pain with endometriosis (*n* = 35), chronic pelvic pain without endometriosis (*n* = 23), and patients with no chronic pelvic pain or endometriosis (*n* = 15). Four women were excluded from the study due to incidental endometriosis with no chronic pelvic pain and a small sample size. Of the women diagnosed with CPP without endometriosis, ten women noted a self-report history of endometriosis. Women who were diagnosed with CPP with endometriosis were then given endometriosis staging based on the American Society for Reproductive Medicine revised classification: stage 1 (*n* = 15), stage II (*n* = 7), stage III (*n* = 2), and stage IV (*n* = 12)

**Fig. 2:**
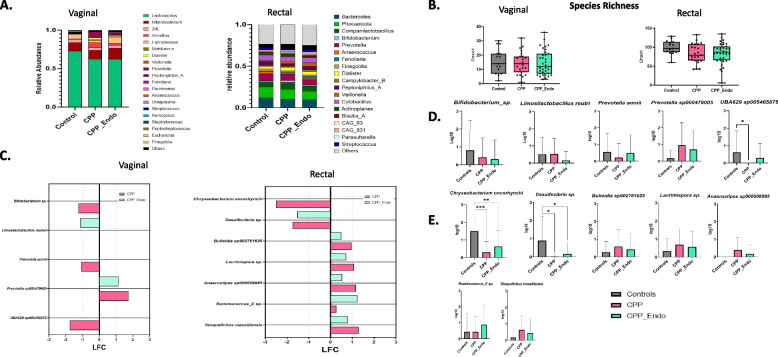
The vaginal and rectal microbiome in chronic pelvic pain, endometriosis, and controls. The rectal microbiome is significantly different between patients diagnosed with chronic pelvic pain and no chronic pelvic pain. **A** Grouped taxa bar plot of genera in the vaginal and rectal microbiomes across diagnosis groups: controls with no chronic pelvic pain (Controls), chronic pelvic pain (CPP), and chronic pelvic pain with endometriosis (CPP-Endo). **B** Chao1 species richness is not significantly different between Controls, CPP, and CPP-Endo in the vaginal microbiome (*p*-value: 0.89226; (ANOVA) *F*-value: 0.11418) or rectal microbiome (*p*-value: 0.10783; (ANOVA) *F*-value: 2.2996). **C** Differentially abundant vaginal and rectal bacterial taxa among CPP and CPP-Endo compared to controls. The differential abundance analysis was performed utilizing ANCOM-BC, visualized taxa with at least 1 log fold change. Other significant taxa are in Supplement Fig. 4, and all *p*-values were Bonferroni false discovery adjusted where *q*-value < 0.05 was significant. **D** Boxplots of vaginal bacteria indicated as significant differentially abundant log10 transformed, where abundance is observed by the diagnosis group. Gray indicates Controls, light pink indicates CPP, and light green indicates CPP-Endo. Additional abundance differences were performed by the Kruskal–Wallis test where * is < 0.05, ** is < 0.01, *** < 0.001, and **** < 0.0001 *p*-value

Patients meeting the criteria for inclusion were asked to complete a questionnaire to evaluate patient history, treatments, and symptoms prior to their planned surgery. Patients were assigned to the chronic pelvic pain (CPP) group if the review of clinical records indicated at least 6 months of pain located in the pelvis. Patients were diagnosed with endometriosis if there was pathological confirmation of endometriosis on excised specimens from the study surgery or if records indicated prior pathologic confirmation. Patients with CPP and no confirmation of endometriosis, either based on negative visual inspection or negative pathology of biopsies, were assigned to the CPP without endometriosis group. One patient in our CPP and endometriosis group was excluded from the microbiome and immunoproteome analysis due to missed specimen collection. Four patients from the control group were found to have pathology-confirmed endometriosis at the time of surgery and were excluded. The final groups for analysis included CPP with confirmed endometriosis (*n* = 35), CPP and no endometriosis (*n* = 23), and controls (*n* = 15) (Fig. 1). Indications for laparoscopy in the control group included sterilization (*n* = 8), uterine fibroids (*n* = 3), abnormal uterine bleeding (*n* = 5), adnexal mass (n=2), malposition of an intrauterine device (*n* = 1), suspected adenomyosis (*n* = 1), and pelvic organ prolapse (*n* = 1). Sensitivity analysis was performed on co-occurring condition groups (AUB/no AUB, fibroids/no fibroids, cysts/no cysts), endometriosis characterization such as stage (stage 1/2 and 3/4), and endometriosis location (peritoneum, multiple sites, other sites, and ovary) (Fig. 2).

### Demographic and clinical analysis

Descriptive statistics were conducted in SAS version 9.4 stratified by patient group (CPP-Endo, CPP alone, and control). Baseline survey variables included age, race, ethnicity, marital status, education, household income, and age at menarche. Body mass index, diagnosis of fibroids, abnormal uterine bleeding, ovarian cysts, and adenomyosis from the patient’s medical records were also included in the descriptive analysis. Mean and standard deviation were calculated for continuous variables, and the number and frequency were calculated for categorical variables. To evaluate differences between the patient groups, Fisher’s exact chi-square (for categorical variables) and analysis of variance (ANOVA) (for continuous variables) tests were performed.

### Sample collection and processing

Enrolled patients underwent sample collection in the operating room after administration of anesthesia but before the pelvic exam, lubricant use, or vaginal prep administration. The vaginal pH was determined using nitrazine paper, and sterile swabs were used to collect samples from the vagina and rectum utilizing an established protocol [33]. To collect the cervicovaginal lavage (CVL), 10 ml of 0.9% sterile saline (Teknova, Hollister, CA) was instilled into the vagina, and a sterile pipette was used to bathe the cervix for 1 min. This lavage fluid was then collected into the pipette and dispensed into a sterile 15-ml conical tube. All samples were labeled and stored in a − 80°C freezer within 1 h of collection. Before downstream analyses, CVLs were thawed on ice and cleared by centrifugation (700 × *g* for 10 min at 4°C). Vaginal swabs were collected using an eSwab collection system with Amies transport medium (COPAN Diagnostics, Murrieta, CA). DNA was extracted from swabs using Dnaeasy PowerSoil kit (Qiagen, Germantown, MD) following the manufacturer’s protocol. CVLs and extracted DNA were aliquoted to avoid multiple freeze–thaw cycles and stored at − 80°C. Blood was observed in 34% (*n* = 25) of vaginal samples.

### 16S rRNA sequencing, processing, and bacterial classification

DNA from clinician-collected vaginal and rectal swabs underwent 16S rRNA gene sequencing. The 16S rRNA gene was amplified using the V4 region with MyFi™ Mix (Bioline Meridian, Cat No. BIO-25050). Golay barcode-tagged forward primer and Earth Microbiome Project (EMP) primers (515F-806R) for reverse primers and sequenced on the Illumina MiSeq benchtop sequencing platform (Illumina, San Diego, CA). The PCR was performed on LightCycler 96 (Roche). Amplicons were quantified using Quant-It PicoGreen dsDNA Assay kit (ThermoFisher Scientific, Cat No. P7589), according to the manufacturer’s protocol. Equal amounts of 240 ng of amplified DNA from each sample were pooled and cleaned using UltraClean PCR Clean-Up Kit (QIAquick PCR Purification Kit, Qiagen, Cat No. 28104). The library was sequenced at the PANDA Core for Genomics and Microbiome Research at the University of Arizona, Steele Children’s Research Centre on MiSeq platform (Illumina).

Microbial DNA sequencing data were processed and analyzed using the QIIME 2 version 2022.2 and demultiplexed using the q2-demux plugin [34]. Human reads were filtered out by aligning to a hg19 reference human [35] genome using bowtie2. The SAM file output was provided by bowtie2 and converted to a BAM file using samstools [36], then converted to a FASTQ file using Bedtools [37]. We utilized DADA2 for quality filtration, trimming to forward 240 and reverse 220 nt, merging, denoising, and generation of amplicon sequence variants (ASVs) utilizing the q2-dada2 plugin [38]. Bacterial taxonomy was assigned to ASVs using the q2-feature-classifier plugin with the classify-sklearn naive Bayes classifier against the Genome Taxonomy Database (GTDB), version r202 [39–42]. Taxonomic class weights were utilizing the pluginq2-clawback [43]. The vaginal classifier was created using methods outlined in Bokulich et al. [44]. The rectal classifier was based on publicly available stool data in Qiita [45]. Sequences that failed to classify at the phylum level were discarded, and ASVs with less than 0.01% relative abundance and 10% prevalence were filtered out before downstream analysis. Rarefaction was applied to avoid introducing library size bias.

### Global microbiome analysis

To analyze the rectal and vaginal microbiome profiles between disease groups (CPP alone, CPP-Endo, controls), co-occurring condition groups, endometriosis characterization groups (stage and location), and blood in sample groups, we utilized MicrobiomeAnalyst 2.0 [46]. The relative abundances of taxa in the groups mentioned above were revisualized as stacked bar plots with Prism 9.0 (GraphPad, San Diego, CA). Taxa were plotted at the genus level, and any taxa with < 0.01% in profile were merged into “Others.” Alpha diversity was computed using the Shannon diversity index and Chao1, and statistical comparisons between groups were performed with T-test/ANOVA tests. Beta-diversity metrics were computed using the Bray–Curtis dissimilarity index, and group comparisons were performed with the nonparametric permutational multivariate analysis of variance (PERMANOVA). Vaginal *Lactobacillus* and *Limosilactobacillus* abundance were compared with Mann–Whitney *U* [47] statistical tests. 

### Differential abundance

ASVs identified at the species level were analyzed utilizing analysis of compositions of microbiomes with bias correction (ANCOM-BC) R package [48] to determine if they were differentially abundant between analysis groups. *p*-values were corrected for multiple comparisons using the false discovery rate (FDR) method, and taxa with a log fold change (LFC) > 1 and a *q*-value < 0.05 were considered significant. Additional confirmatory analysis was conducted utilizing the HeatTree method [49] and Wilcoxon rank sum test by MicrobiomAnalyst 2.0 [46].

### Quantification of soluble immune proteins

CVL samples were utilized for immunoproteome analysis as previously described [50]. Briefly, levels of 41 proteins were measured in CVL samples using the Milliplex MAP Magnetic Bead Immunoassays: Human Cytokine Chemokine Panel 1 (Millipore, Billerica, MA) following the manufacturer’s protocols [50]. Data was collected using a Bio-Plex 200 instrument and analyzed using Manager 5.0 software (Bio-Rad, Hercules, CA). Concentration values below the detection limit were substituted with 0.5 of the minimum detectable concentration provided in the manufacturer’s protocol. Protein concentrations were log10 transformed and auto-scaled (mean-centered and divided by the standard deviation) before bioinformatics analysis.

### Immune protein analysis

Hierarchical clustering analysis was performed to observe relationships between protein biomarker levels and patient and sample metadata. Unsupervised hierarchical clustering was performed with ClustVis [51] utilizing Euclidean distance and Ward linkage. The statistical differences in the distribution of patient-related factors between clusters were assessed using Fisher’s exact or chi-square test.

Principal component analysis was performed to reduce the observed variables into principal components that account for most of the variance in the observed variables to observe trends. Principal components (PC1 and PC2) were analyzed to observe differences among groups using the Kruskal–Wallis and Dunn’s multiple comparisons tests. PCA score analysis was performed.

Differences in the protein levels among disease groups, endometriosis characteristics, and co-occurring conditions were tested using two-sample *t*-tests fold-change analysis and corrected using the false discovery rate method. Differences in mean protein levels and *q*-values were assessed by MetaboAnalyst 5.0 [52]. Protein immune markers with *q*-value < 0.05 were considered significant.

### Immune-microbiome correlation

Spearman’s rank correlation analysis was performed to investigate the association of immune protein levels with significant vaginal taxa identified with ANCOM-BC [48]. A correlation matrix was computed using correlation coefficients with *p*-values utilizing Prism 9.0 (GraphPad, San Diego, CA) and graphically presented as a heat map utilizing ClustVis [51]. *p*-values < 0.05 were considered significant.

## Results

### Patient demographics

In this pilot study, we investigated vaginal and rectal microbiome and immunoproteomic differences between patients with CPP and endometriosis, patients with CPP alone, and patients with no CPP or endometriosis. We recruited women undergoing laparoscopy (*n* = 81) from 02/07/2019 to 11/29/2021 and collected vaginal and rectal swabs and cervicovaginal lavage samples prior to surgery for downstream analysis (Fig. 1). The women were stratified according to whether they were seeking care for pelvic pain lasting for more than 6 months, including CPP alone (*n* = 23) and CPP with endometriosis (CPP-Endo) (*n* = 36). As a comparison group, we also included patients undergoing surgery who had no diagnosis of CPP or endometriosis (*n* = 15). The confirmed endometriosis diagnosis was based on postoperative histopathological confirmation (Fig. 1).

CPP-Endo, CPP alone, and control groups were similar in age (mean age 35 years) (Table 1). Our patient cohort was predominately White (*n* = 59/74) (Table 1), which may be due to the underreporting of benign gynecologic conditions in other racial and ethnic minority groups [53]. Regarding ethnicity, patients with CPP-Endo had more patients who were non-Hispanic women (80%) compared to CPP alone (47.8%) and controls (33.3%) (*p* = 0.004) (Table 1). BMI, marital status, education, hormonal treatment, and age at menarche did not differ between groups. However, income levels differed between groups, with CPP-Endo reporting higher incomes than CPP without endometriosis and the controls (*p* = 0.05) (Table 1). Several co-occurring benign gynecologic conditions were noted but did not differ between groups (Table 1). Specifically, we observed diagnoses of pathology-confirmed adenomyosis (*n* = 6), pathology-confirmed fibroids (*n* = 22), preoperative diagnosis of abnormal uterine bleeding (AUB) (*n* = 26), and pre-operative or intraoperative findings of an ovarian cyst (*n* = 29) (Additional file 1: Fig. S1). Symptoms of AUB including irregular menstruation (*n* = 14) and heavy menstruation (*n* = 17) were also reported.

**Table 1:** Patient demographics and characteristics of chronic pelvic pain cohort. *p*-values were calculated using analysis of variance (ANOVA) for continuous values and chi-square test for categorical values. The overall difference was significant at *p* < 0.05. Descriptive statistics stratified by patient group (CPP with endometriosis, CPP without endometriosis, and Control). Variables that were included were age, race, ethnicity, marital status, education, household income, age at menarche, gravidity, parity, body mass index, hormonal treatment, diagnosis of fibroids, abnormal uterine bleeding, ovarian cysts, and adenomyosis. *CPP* chronic pelvic pain, controls, *SD* standard deviation

	**CPP with endometriosis** **(*n* = 36)**	**CPP without endometriosis** **(*n* = 23)**	**Surgical control** **(*n* = 15)**	***p*-value**^**1**^
Age, mean (S.D.)	34.7 (8.8)	36.5 (7.2)	40.6 (8.2)	0.07
Race				0.90
White	29 (80.6)	18 (78.3)	12 (80.0)	
Non-White	7 (19.4)	4 (17.4)	3 (20.0)	
Missing	-	1 (4.4)	-	
Ethnicity				*0.004*
Hispanic or Latino	6 (16.7)	9 (39.1)	9 (60.0)	
Not Hispanic or Latino	29 (80.6)	11 (47.8)	5 (33.3)	
Missing	1 (2.8)	3 (13.0)	1 (6.7)	
Body mass index				0.3
< 25	13 (36.1)	5 (21.7)	1 (6.7)	
25–28.9	6 (16.7)	4 (17.4)	4 (26.7)	
≥ 30	17 (47.2)	14 (60.9)	10 (66.7)	
Missing	-	-	-	
Marital status				1.0
Single	14 (38.9)	9 (39.1)	6 (40.0)	
In a relationship	20 (55.6)	13 (56.5)	9 (60.0)	
Missing	2 (5.6)	1 (4.4)	-	
Education				0.3
No college	19 (52.8)	14 (60.9)	11 (73.3)	
College and above	15 (41.7)	4 (17.4)	1 (6.7)	
Missing	2 (5.6)	5 (21.7)	3 (20.0)	
Household income				*0.05*
< 50,000	11 (30.6)	10 (43.5)	9 (60.0)	
50,000 +	19 (52.8)	6 (26.1)	3 (20.0)	
Missing	6 (16.7)	7 (30.4)	3 (20.0)	
Age menarche, mean (S.D.)	11.7 (1.6)	11.3 (2.4)	11.1 (1.8)	0.6
*N* missing values for age menarche	7	10	6	
Fibroids confirmed at time of surgery (*N* yes, %)	12 (33.3)	6 (26.1)	4 (26.7)	0.8
Abnormal uterine bleeding confirmed at time of surgery (*N* yes, %)	12 (33.3)	9 (39.1)	5 (33.3)	0.9
Ovarian cysts confirmed at time of surgery (*N* yes, %)	18 (50.0)	8 (34.8)	3 (20.0)	0.1
Adenomyosis confirmed at time of surgery (*N* yes, %)	2 (5.6)	3 (13.0)	1 (6.7)	0.7
Hormonal treatment within 1 month leading up to surgery				
Progestin	13 (36.11)	7 (30.43)	6 (40.0)	0.86
Combined estrogen/progestin	7 (19.44)	5 (21.74)	1 (6.67)	0.52
GnRH ant/agonist	1 (2.78)	1 (4.35)	-	0.31
Other	-	-	-	-
None	15 (41.67)	11 (47.83)	8 (53.33)	0.75
Estrogen	-	-	-	
Testosterone	-	-	-	
Combined hormonal medication use				0.75
No	15 (41.67)	11 (47.83)	8 (53.33)	
Yes	21 (58.33)	12 (52.17)	7 (46.67)	
Vaginal pH, mean (S.D.)	5.4 (1.2)	4.9 (0.9)	5.1 (1.2)	0.4

*p*-values were calculated using chi-square Fisher’s exact for categorical variables and ANOVA for continuous variables. Missing values were excluded

### Vaginal and rectal microbiome and CPP and CPP with endometriosis

When investigating the overall community composition differences between diagnosis groups at the genus level, we did not observe global differences between groups (Fig. 2A). *Lactobacillus* predominance in the vagina, which is often associated with homeostasis in the vagina, was observed in all three diagnosis groups (Fig. 2A). Investigation at the species level indicated *Lactobacillus jensenii* was more abundant in the CPP-Endo compared to CPP alone (*p* < 0.05) and controls (*p* < 0.05) (Additional file 1: Fig S2). However, when investigating the overall composition, no differences in species richness and evenness in the vaginal and rectal samples were observed (Fig. 2B, Additional file 1: Fig S3). However, rectal samples from controls were slightly more diverse than CPP alone and CPP-Endo (Fig. 2B, Additional file 1: Fig. S3). There were no statistically significant differences in beta diversity between diagnosis groups in the vaginal or rectal samples (Additional file 1: Fig. S3).

To identify microbial signatures that are unique to patients diagnosed with CPP alone and CPP-Endo compared to controls, we applied ANCOM-BC [48] (Fig. 2C, Additional file 1: Fig. S4, Additional file 2: Table S1). Investigation of vaginal samples revealed that 27 vaginal species were statistically significantly enriched or depleted in CPP alone (18) and CPP-Endo (17) compared to controls (Additional file 1: Fig. S4). The most differentially abundant Log fold change (LFC) of 1, in CPP alone and CPP-Endo compared to controls was an enrichment of *Prevotella* sp000479005 (*q* < 0.00001, *q* < 0.00001) (Fig. 2C, D). Patients with CPP alone also had a depletion of *UBA629* sp005465875 (*Lachnospiraceae* family member) (*q* < 0.00001), *Prevotella amnii* (*q* < 0.00001), and an unclassified *Bifidobacterium* species (*q* < 0.00001) compared to controls (Fig. 2C, D), while CPP-Endo had a depletion of *Limosilactobacillus reuteri* (*q* < 0.0001) compared to controls (Fig. 2C, D). We also investigated differential abundance, comparing CPP-Endo to CPP alone (Additional file 1: Fig. S5, Additional file 2: Table S2). In this analysis, CPP-Endo had ten enriched and seven depleted species compared to CPP alone. Of note, *Fusobacterium animalis,* a related species in genus *Fusobacterium* that has been recently associated with endometriosis pathogenesis [54], was depleted in CPP-Endo compared to CPP alone, in our cohort with a LFC of − 0.63 (Additional file 1: Fig. S5). However, only *Streptococcus anginosus* (*q* < 0.0001) was noted to be the most enriched in the CPP-Endo compared to CPP alone, with an LFC of 1 (Additional file 1: Fig. S5).

Analysis of the rectal microbiome from the rectal samples revealed 59 species that were differentially abundant when comparing CPP alone (41 species, 15 depleted, 26 enriched) and CPP-Endo (42 species, 10 depleted, 32 enriched) to controls (Fig. 2C and Additional file 1: Fig. S4, Additional file 3: Table S11). The most differentially abundant rectal species identified were an unclassified *Desulfovibrio* species (*q* < 0.00001) depleted in both CPP alone and CPP-Endo compared to controls (Fig. 2C, D). Additionally, *Chryseobacterium oncorhynchi* (*q* < 0.00001) was significantly depleted in CPP alone compared to controls with an LFC of − 2.51. Further shared enrichment of *Stoquefichus massiliensis* (*q* < 0.00001), a *Ruminococcus E* species (*q* < 0.00001), *Anaerostipes* sp000508985 (*q* < 0.00001), an unclassified *Lacrimispora* species (*q* < 0.00001), and *Bulleidia* sp902761625 (*q* < 0.00001) were observed in CPP alone and CPP-Endo compared to controls (Fig. 2C, D). When comparing CPP-Endo to CPP alone, there were 35 differentially abundant species, all of which were depleted in CPP-Endo (Additional file 3: S12). However, no rectal species were highly abundant across samples, meaning with below a LFC of 1. The closest bacterial species, at a depletion of − 0.84, was *Anaerosacchariphilus* sp002160765.

### Immune markers associated with chronic pelvic pain

**Fig. 3:**
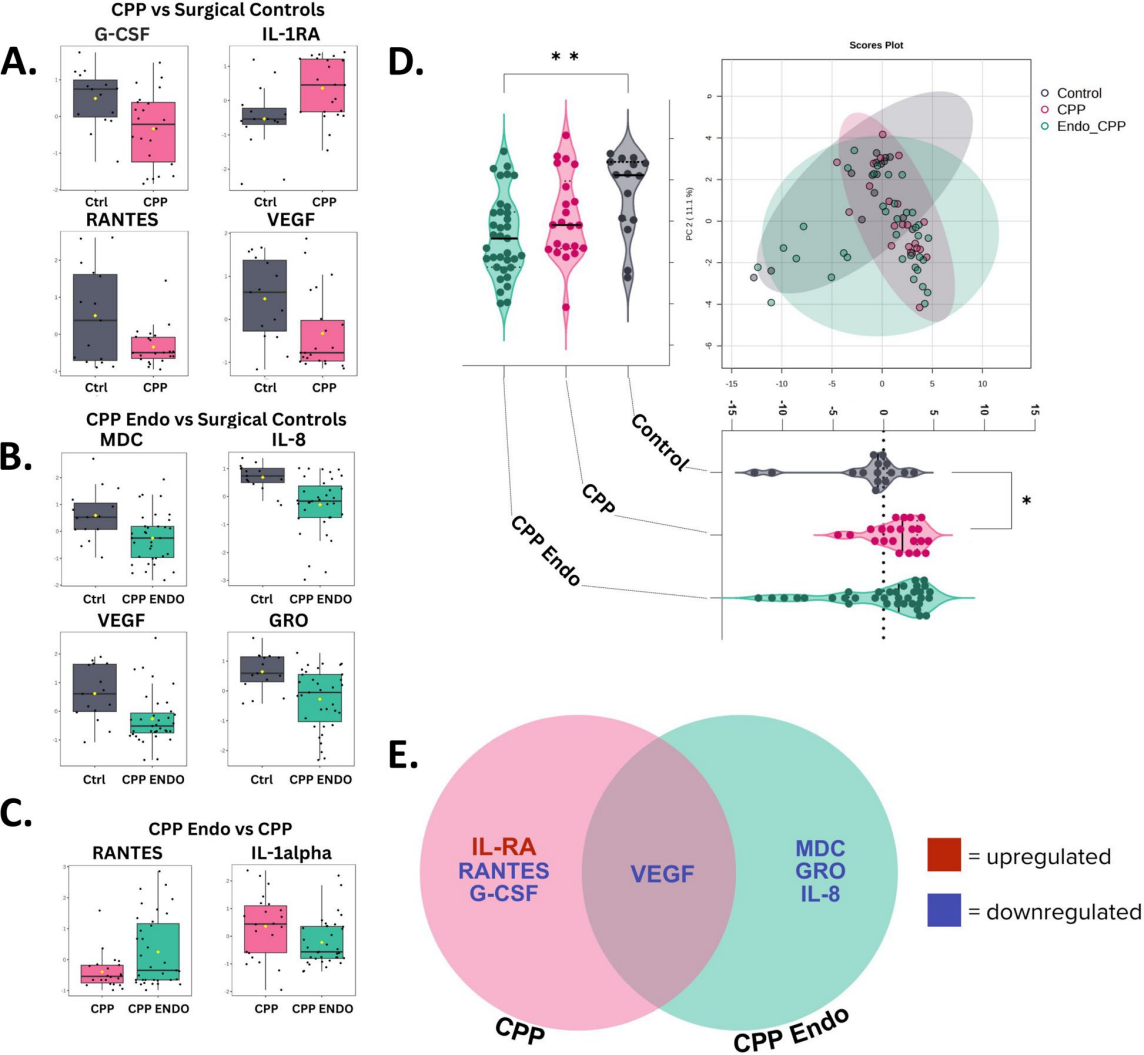
Cervicovaginal lavage immune markers for chronic pelvic pain and chronic pelvic pain with endometriosis. Cervicovaginal levels of protein biomarkers in patients vary based on disease group. A volcano plot analysis was used to assess differences in the immune protein levels among patients based on the diagnosis group. Statistical significance was determined using a two-sample *t*-test with the false discovery rate (FDR) correction. Proteins with *q* < 0.05 were considered significant.** A** Proteins G-CSF, RANTES, and VEGF were significantly downregulated, and IL-1Ra was upregulated across diagnosis groups: controls with no chronic pelvic pain (Ctrl) in gray and chronic pelvic pain (CPP) in pink, **B** proteins MDC, IL-8, GRO, and VEGF were significantly downregulated across diagnosis groups: controls with no chronic pelvic pain (Ctrl) in gray and chronic pelvic pain with endometriosis (EC) in green. **C** Protein IL-1alpha was significantly downregulated, and RANTES was upregulated across diagnosis groups when *p*-value analysis was performed. FDR correction showed no significant markers: chronic pelvic pain (CPP) in pink and chronic pelvic pain with endometriosis (EC) in green. **D** A principal component analysis (PCA) of the diagnosis groups shows distinct differences in diagnosis groups. PC 1 shows a significant difference between controls with no chronic pelvic pain in gray and chronic pelvic pain in pink. PC 2 shows a significant difference between controls with no chronic pelvic pain in gray and chronic pelvic pain with endometriosis in green. Gray indicates controls, pink indicates CPP, and green indicates CPP-Endo. Additional differences between disease groups were performed by ANOVA test where * is denoted as < 0.05, ** is denoted as < 0.01, *** < 0.001, and **** < 0.0001 *p*-value. **E** A Venn diagram showcasing shared and unique markers that were identified with the volcano plot analysis between the diagnosis groups

To study immunoproteomic differences between patients with CPP-Endo, CPP alone, and controls, we investigated the levels of 41 soluble proteins in CVL samples, including cytokines, chemokines, growth factors, and immune checkpoints. To depict global protein profiles of samples between diagnosis groups, we performed a principal component analysis that explained 43.8% (PC1) and 11.1% (PC2) variance of the immune data. These principal components were different between all diagnosis groups (*p* < 0.05) (Fig. 3D). The analysis also revealed that immune profiles differed between CPP alone and controls (*p* < 0.05) as well as CPP-Endo and controls (*p* < 0.01) (Fig. 3D).

**Fig. 4:**
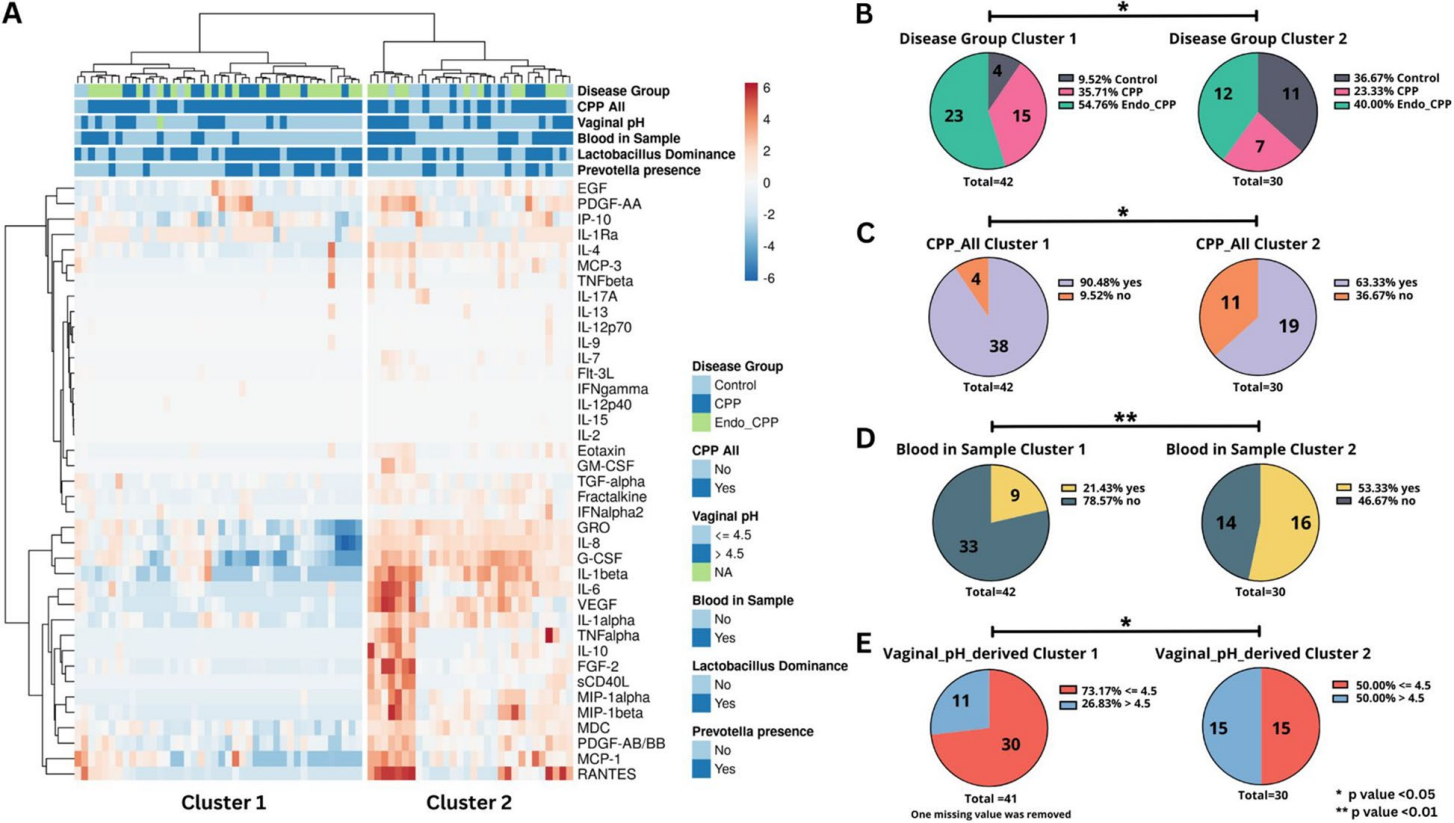
Hierarchical clusters of immune and microbiome profiles indicate unique global profiles from patients with chronic pelvic pain with and without endometriosis. Cervicovaginal protein levels are associated with the disease groups. **A** heatmap reflects relative levels of proteins in cervicovaginal lavages (CVL) across all the samples (*n* = 72). Data were mean-centered and log-transformed. Hierarchical clustering was based on Euclidean distance and Ward linkage. Two immune markers were removed due to being constant, IL-3 and IL-5. The analysis revealed two distinct clusters based on the immune profiles of this cohort. Pie charts show significant differences observed in distribution between immune clusters 1 and 2 for **B** disease groups, **C** CPP status, **D** vaginal pH, and **E** blood in the sample were significantly different between the clusters. *p*-values were calculated using Fisher’s exact test or chi-square test. *p*-values < 0.05 were significant, where * is denoted as < 0.05, ** is denoted as < 0.01, *** < 0.001, and **** < 0.0001 *p*-value

To identify immunological markers that were distinct between diagnosis groups, we utilized a two-sample *t*-test with the false discovery rate (FDR) correction (*p* < 0.05; FC > 2). We observed three immune markers between CPP alone and controls that were decreased, granulocyte colony-stimulating factor (G-CSF) (*q* = 0.049), regulated on activation, normal T cell expressed and secreted (RANTES) (*q* = 0.049), and vascular endothelial growth factor (VEGF) (*q* = 0.049), while interleukin-1 receptor antagonist (IL-1Ra) (*q* = 0.049), was elevated (Fig. 3A, E, Additional file 4: Table S20). Four markers, macrophage-derived chemokine (MDC) (*q* = 0.05), interluekin-8 (IL-8) (*q* = 0.037), VEGF (*q* = 0.044), and growth-related oncogene (GRO) (*q* = 0.042), were decreased when comparing CPP-Endo to controls (Fig. 3B and E, Additional file 4: Table S21). Interleukin-1 alpha (IL-1⍺) (*q* = 0.031) was decreased, and RANTES (*q* = 0.017) was elevated when comparing patients with CPP alone to CPP-Endo (Fig. 3C, E, Additional file 4: Table S22). VEGF was decreased in both CPP alone and CPP-Endo (Fig. 3E). Unsupervised hierarchical clustering based on immune markers revealed two distinct clusters (Fig. 4B). Differences in clinical variables in immune cluster 1 included a higher prevalence of CPP-Endo (*p* < 0.05), less observed blood within the sample (*p* < 0.01), and lower vaginal pH (*p* < 0.05) compared to immune cluster 2 (Fig. 4B and Additional file 1: Fig. S6). Differential immune analysis of the clusters revealed 35/41 statistically significant differences, excluding IL-12 p40, interferon γ-induced protein 10 (IP-10), IL-9, IL-5, IL-2, and IL-1Ra (Additional file 1: Fig. S7). This data indicates that there are some shared immune profiles of CPP, but immune differences may also be attributed to the etiology of CPP, vaginal pH as a proxy for vaginal dysbiosis, and blood remaining in the sample.

### Endometriosis characteristics have defined microbial signatures

To investigate if there were specific microbial and immunoproteomic differences between revised rASRM endometriosis stage [52] and location, which has been recently investigated to better understand the etiology of endometriosis and its relation to symptoms such as pain [55, 56], we conducted a sub-analysis of the CPP-Endo group. The endometriosis stage was dichotomized into stages 1/2 and 3/4 since most patients were either stage 1 or stage 4. Endometriosis location microbial diversity indicated there were no differences in species richness, evenness, or beta diversity based on the endometriosis stage or endometriosis location in vaginal samples (Additional file 1: Fig. S8 and S9). Investigation of rectal samples revealed a similar trend for endometriosis location, but species richness was close to statistical significance (*p* = 0.06), and species evenness (*p* = 0.04) was statistically significantly higher in endometriosis stage 1/2 compared to stage 3/4 (Additional file 1: Fig. S8 and S9). No rectal differences were observed for endometriosis location.

**Fig. 5:**
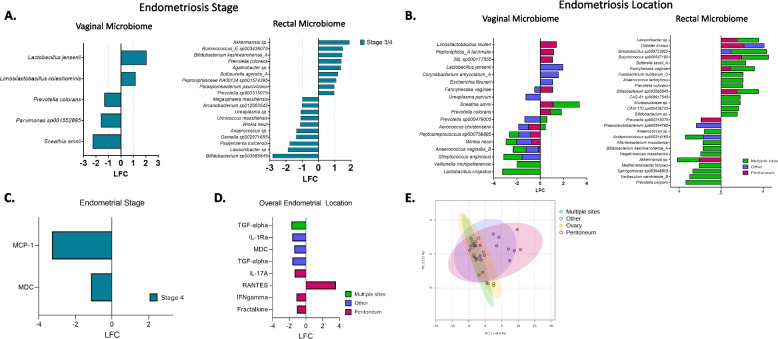
Endometriosis characteristics reveal distinct microbial and immune profiles. Endometriosis characteristics such as stage and location can be differentiated by vaginal and rectal microbiome. **A** Differentially abundant vaginal and rectal bacterial taxa diverging bar plot among ASRM revised classification stages 3/4 (blue) compared to 1/2. **B** Differentially abundant vaginal and rectal bacterial taxa diverging bar plot of the site of endometriosis detected during surgery of peritoneum (pink); other (purple) which included locations of the bladder, vagina, bowel, and lymph node; and multiple locations (green) which we defined as finding endometriosis in more than one determined location during surgery. These locations were compared to the most prevalent location site of the ovary, sometimes referred to as endometrioma. The differential abundance analysis was performed utilizing ANCOM-BC, visualized taxa with at least 1 log fold change; other significant taxa are in supplement Fig. 12, and all *p*-values were Bonferroni false discovery adjusted where *q*-value < 0.05 was significant. **C** Cervicovaginal levels of protein biomarkers in patients vary. A volcano plot analysis was used to assess differences in the immune protein levels among patients based on the diagnosis group. Statistical significance was determined using a two-sample *t*-test with the false discovery rate correction. Proteins with *q* < 0.05 were considered significant. Protein biomarker diverging bar plot among ASRM revised classification stages 3/4 (blue) compared to 1/2. **D** Protein biomarker diverging bar plot of the site of endometriosis detected during surgery of peritoneum (pink); other (purple); and multiple locations (green), which we defined as finding endometriosis in more than one determined location during surgery. These locations were compared to the site of the ovary. The protein biomarker analysis was performed utilizing a *t*-test, visualized markers where *q*-value < 0.05 was significant. **E** PCA plot of immune markers by endometriosis location sites. Utilizing the Kruskal–Wallis test, no significant difference between endometrial locations was found

Vaginal *Lactobacillus* at the genus level did not show differences between endometriosis stage 1/2 and 3/4; however, differential abundance analysis observed that *L. jensenii* (*q* < 0.00001) and *Limosilactobacillus coleohominis* (*q* < 0.0001) were differentially abundant in stage 3/4 (Fig. 5A, Additional file 1: Fig. S10 and S11). Five bacteria were enriched in stage 3/4, including *Peptoniphilus B duerdenii* (*q* < 0.0001), *Varibaculum cambriense B* (*q* < 0.0001), a *Campylobacter B* species(*q* < 0.0001), *Winkia neuii* (*q* < 0.0001), and *Anaerococcus vaginalis_B* (*q* < 0.0001) (Fig. 5A and Additional file 1: Fig. S11). At the same time, 11 bacterial vaginosis-associated bacteria (BVAB) were depleted in stage 3/4 compared to stage 1/2, including *Sneathia amnii* (*q* < 0.0001), *Parvimonas sp001552895* (*q* < 0.0001), *Prevotella colorans* (*q* < 0.0001), *Prevotella sp000479005* (*q* < 0.0001), *Corynebacterium sp.*(*q* < 0.0001), *Fannyhessea vaginae A* (*q* < 0.0001), *Peptoniphilus A lacrimalis* (*q* < 0.0001), *Anaerococcus marasmi* (*q* < 0.0001), *Ezakiella coagulans* (*q* < 0.0001), *Mobiluncus mulieris* (*q* < 0.0001), and *Prevotella disiens* (*q* < 0.0001) (Fig. 5A, Additional file 1: Fig. S11, Additional file 2: Table S3). Rectal bacterial differences were also observed in 53 species, with 23 enriched and 30 depleted, comparing stages 3/4 to 1/2. Of these, the most enriched in stage 3/4 were *Prevotella sp900315075* (*q* < 0.0001), *Parasporobacterium paucivorans* (*q* < 0.0001), *Peptoniphilaceae KA00134 sp001574395* (*q* < 0.0001), *Buttiauxella agrestis A* (*q* < 0.0001), *Agathobacter* sp. (*q* < 0.0001), *Prevotella colorans* (*q* < 0.0001), *Bifidobacterium kashiwanohense A* (*q* < 0.0001), *Ruminococcus_E sp003438075* (*q* < 0.0001), and *Akkermansia* sp. (*q* < 0.0001) (Fig. 5A and Additional file 1: Fig. S11, Additional file 3: Table S13). Most depleted were *Bifidobacterium* sp003585845 (*q* < 0.0001), *Lawsonibacter* sp. (*q* < 0.0001), *Pauljensenia turicensis* (*q* < 0.0001), *Gemella* sp002871655 (*q* < 0.0001), *Anaerococcus* sp. (*q* < 0.0001), *Winkia neuii* (*q* < 0.0001), *Urinicoccus massiliensis* (*q* < 0.0001), *Ureaplasma* sp. (*q* < 0.0001), *Arcanobacterium* sp012563545(*q* < 0.0001), and *Megasphaera massiliensis* (*q* < 0.0001) (Fig. 5A and Additional file 1: Fig. S11, Additional file 3: Table S13).

Although staging is related to endometriosis location, we aimed to investigate further bacterial differences among sites of endometriosis lesions: peritoneum, ovary, multiple locations, and other locations (bladder, bowel, etc.). Our analyses revealed that 28 vaginal bacteria differed between locations 14 enriched and 14 depleted, where other locations had the most differences, followed by peritoneum and then multiple locations (Fig. 5B and Additional file  1: Fig. S12, Additional file 2: S4). The most depleted by all three sites compared to the ovary were *Anaerococcus vaginalis B* (*q* < 0.0001), *Winkia neuii* (*q* < 0.0001), *Peptostreptococcus* sp000758885 (*q* < 0.0001), and *Aerococcus christensenii* (*q* < 0.0001) (Fig. 5B). *S. anginosus* (*q* < 0.0001) was depleted in both other locations and multiple locations compared to the ovary (Fig. 5B). *Lactobacillus crispatus* (*q* < 0.0001) and *Veillonella montpellierensis* (*q* < 0.0001) were only depleted when multiple locations were compared to the ovary (Fig. 5B and Additional file 1: Fig. S12). *Ureaplasma parvum* (*q* < 0.0001) was only depleted in other locations compared to the ovary (Fig. 5B). *Prevotella colorans* (*q* < 0.0001) and *Sneathia amnii* (*q* < 0.0001) were enriched in both the peritoneum and multiple location sites compared to the ovary (Fig. 5B). The most differentially enriched for only peritoneum location compared to ovary was 28L (*Megsphaera lornae*) (*q* < 0.0001), *Peptoniphilus A lacrimalis* (*q* < 0.0001), and *Limosilactobacillus reuteri* (*q* < 0.0001) (Fig. 5B). *Escherichia flexneri* (*q* < 0.0001), *Corynebacterium amycolatum A* (*q* < 0.0001), and *L. jensenii* (*q* < 0.0001) were only enriched in other sites compared to the ovary (Fig. 5B and Additional file 1: Fig. S12). Interestingly, *Fannyhessea vaginae* (*q* < 0.0001) was enriched in the peritoneum and depleted in other sites compared to the ovary (Fig. 5B). While *Prevotella* sp000479005, which was associated with CPP alone and CPP-Endo, was enriched in multiple locations but depleted in other sites (Fig. 5B). Thus, these two organisms may have a more local environmental role in disease than bacteria associated with disease in other locations, which may play more systemic roles.

Our analyses of the rectal microbiome also observed differences between endometriosis locations. Rectal bacterial differences were 31 enriched and 28 depleted, 59 total; where multiple locations (34, 17, 17, respectively) had the most differences, followed by peritoneum (25, 15, 10, respectively), and then other locations (23, 10, 13, respectively) (Fig. 5B and Additional file 1: Fig. S12, Additional file 3: Table S14). *Akkermansia* sp. (*q* < 0.0001) depletion was shared between all three sites compared to the ovary (Fig. 5B). *Acidaminococcus* sp900314165 (*q* < 0.0001) was depleted in both other and multiple locations compared to the ovary (Fig. 5B). While only *Prevotella* sp900315075 (*q* < 0.0001) was depleted in the peritoneum, *Phascolarctobacterium* sp900544795 (*q* < 0.0001) was depleted in only other sites. Multiple locations had the most unique number of depleted organisms compared to ovary such as *Anaerococcus* sp. (*q* < 0.0001), *Negativicoccus massiliensis* (*q* < 0.0001), *Bifidobacterium kashiwanohense A* (*q* < 0.0001), *Alterileibacterium massiliense* (*q* < 0.0001), *Prevotella corporis* (*q* < 0.0001), *Varibaculum cambriense B* (*q* < 0.0001), *Sphingomonas sp003946805*(*q* < 0.0001), and *Mediterraneibacter torques* (*q* < 0.0001) (Fig. 5B). *Dialister invisus* (*q* < 0.0001) was enriched in the peritoneum and other samples, while *Streptobacillus* sp009733925 (*q* < 0.0001) was enriched in multiple locations and other locations compared to the ovary (Fig. 5B). *Bifidobacterium* sp003585845 (*q* < 0.0001), *Fannyhessea vaginae* (*q* < 0.0001), *Lawsonibacter* sp. (*q* < 0.0001), and *Butyricicoccus* sp900547195 (*q* < 0.0001) were enriched in multiple locations and peritoneum (Fig. 5B). *Bifidobacterium* sp. (*q* < 0.0001), CAG-170 sp000436735 (*q* < 0.0001), *Muribaculaceae* sp. (*q* < 0.0001), *CAG-81 sp009917545* (*q* < 0.0001), *Prevotella colorans* (*q* < 0.0001), *Anaerococcus lactolyticus* (*q* < 0.0001), *Fusobacterium nucleatum D* (*q* < 0.0001), and *Sutterella seckii A* (*q* < 0.0001) were uniquely enriched in rectal samples of multiple endometriosis sites (Fig. 5B).

### Endometriomas exhibit a distinct inflammatory signature compared to other endometriosis sites

Since we observed differences in endometriosis characteristics in microbial profiles, we utilized a two-sample *t*-test on the immunoproteomic profiles of the endometriosis stage and site of endometriosis. Two markers differentiated stage 3/4 and 1/2, which were MDC (*q* = 0.017) and monocyte chemoattractant protein-1 (MCP-1) (*q* = 0.042) which were decreased in stage 3/4 (Fig. 5C, Additional file 4: Table S23). Eight immune markers were significantly altered based on the site of endometriosis compared to the most observed site, endometriomas (Additional file 4: Tables S24, S25, S26). The peritoneum had a depleted level of fractalkine (*q* = 0.010), IFNγ (*q* = 0.03), and IL-17A (*q* = 0.033). The only elevated level was RANTES (*q* = 0.032), which was observed in the peritoneum compared to endometriomas (Fig. 5D, E). Multiple lesion sites and other sites, such as the rectum, had a decreased level of transforming growth factor alpha (TGFα) (*q* = 0.006, *q* = 0.001, respectively). In addition, other sites had a depleted level of MDC (*q* = 0.014) and IL-1Ra (*q* = 0.046) (Fig. 5D, E). Thus, the ovary has the most alterations in immune factors compared to other endometriosis locations.

### Co-occurring conditions may drive microbial signatures observed with CPP

Given the presence of co-occurring gynecologic conditions within our diagnosis groups, we performed a sub-analysis of the vaginal and rectal samples based on the presence of ovarian cysts, fibroids, and AUB, including heavy and irregular menstrual bleeding. Adenomyosis co-occurs with endometriosis ranging from 27 to 40% [57]. Our cohort with histologically confirmed methods only identified six patients with co-morbid endometriosis and adenomyosis. Therefore, downstream analyses were unable to be performed. There were no differences in species richness or evenness based on the presence or absence of ovarian cysts, fibroids, AUB, or irregular or heavy menstrual bleeding (Additional file 1: Fig. S13 and S14). There were no differences in beta diversity between the co-occurring conditions (Additional file 1: Fig. S15).

**Fig. 6:**
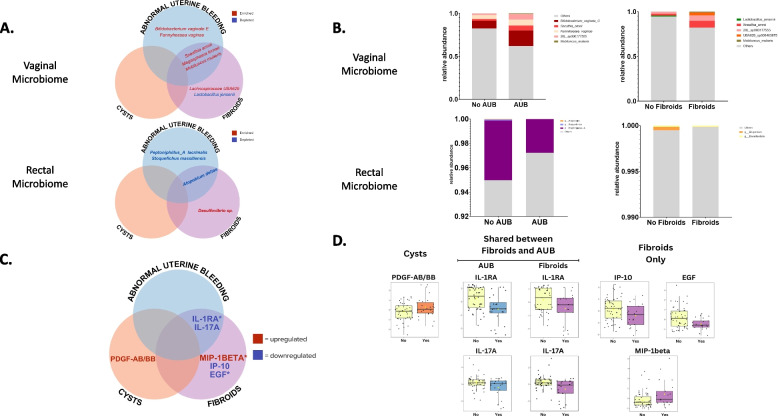
Co-occurring conditions of chronic pelvic pain reveal unique microbial and immune signatures. Abnormal uterine bleeding and fibroids had differences in bacterial vaginosis-associated bacteria and depletion of cytokines and growth factors. **A** Venn diagram of vaginal and rectal bacteria that were most differentially abundant of co-occurring conditions: AUB and no AUB (blue circle), fibroids and no fibroids (purple circle), ovarian cysts and no ovarian cysts (orange circle). Bacterial names in the co-occurring condition group were identified as enriched (red) or depleted (blue). Labeled were taxa with at least 1 log fold change, other significant taxa are in additional data file 1, and all *p*-values were FDR adjusted where *q*-value < 0.05 was significant. No bacteria that fit these criteria were identified for diagnosis of ovarian cysts compared to no ovarian cysts. **B** Grouped relative abundance stacked bar plots for vaginal and rectal differentially abundant bacteria in no AUB vs. AUB and no fibroids vs. fibroids. Rectal stacked bar plots were zoomed in to 92% and above relative abundance due to the large diversity of the rectal microbiome to visualize differences between co-occurring condition groups.** C** Venn diagram of immune proteins that were significantly upregulated or downregulated of histologically confirmed co-occurring conditions groups. Proteins in the co-occurring condition group were identified as enriched (red) or depleted (blue). The analysis was performed utilizing volcano plot analysis labeled proteins with at least 1 log fold change. *p*-values were Bonferroni adjusted where *q*-value < 0.05 was significant are indicated by an asterisk (*). **D** Graphs of the relative concentration levels of proteins between the groups of histologically confirmed co-occurring conditions groups of AUB (blue) and no AUB (yellow), fibroids (purple) and no fibroids (yellow), ovarian cysts (orange) and no ovarian cysts (yellow). *p*-values that were not Bonferroni adjusted are shown where *p*-value < 0.05 was significant

There were no differential vaginal or rectal bacteria identified in patients with ovarian cysts (Additional file 2: Table S5, Additional file 3: Table S15). Differential analysis revealed patients with fibroids had increased *L. jensenii* (*p* < 0.05) (Additional file 1: Fig. 16) as well as BVAB in the vaginal samples including *Lachnospiraceae UBA629* (*q* < 0.0001), *Megasphaera lornae* (*q* < 0.0001), *Mobiluncus mulieris* (*q* < 0.0001), and *Sneathia amnii* (*q* < 0.0001) (Fig. 6A, B, Additional file 1: Fig. S17 and S19, Additional file 2: Table S6). Patients with AUB had an increase of the same BVAB in addition to vaginal *Fannyhessea vaginae* (*q* < 0.0001) (Fig. 6A, B, Additional file 1: Fig. S17 and S19, Additional file 2: Table S7). In rectal samples, *Desulfovibrio* was uniquely enriched in patients with fibroids, while several species were decreased in patients with AUB, including *Stoquefichus massiliense* (*q* < 0.0001) and *Peptoniphilus lacrimalis (q* < 0.0001) (Fig. 6A, B, Additional file 1: Fig. S18 and S20, Additional file 3: Tables S16 and S17). *Atopobium deltae* was depleted in patients with fibroids and AUB (Fig. 6A, B, Additional file 1: Fig. S18 and S20, Additional file 3: Tables S16 and S17).

Due to the strong signatures observed with BVAB and AUB, further analysis was conducted on specific types, such as heavy menstrual and irregular bleeding. Heavy menstrual bleeding had three enriched vaginal bacteria, of which *M. lornae* (*q* < 0.0001) was the most abundant (Additional file 1: Fig. S21 and S23). However, 18 vaginal bacteria were depleted, with *Limosilactobacillus reuteri,* formerly *Lactobacillus reuteri* (*q* < 0.0001) and *L. jensenii* (*q* < 0.0001) being the most depleted (Additional file 2: Table S8). Irregular menstrual bleeding revealed 14 enriched taxa, with *Bifidobacterium vaginale C* (*q* < 0.0001) being the most enriched (Additional file 1: Fig. S22 and S24). While 15 were depleted, including *Fusobacterium animalis* (*q* < 0.0001), none were largely depleted (Additional file 1: Fig. S23, Additional file 2: Table S9). Rectal samples indicated heavy menstrual bleeding had no largely enriched bacteria but did have depleted *Faeclimonas sp900551895* (*q* < 0.0001) (Additional file 1: Fig. S23, Additional file 3: Table S18)*.* While irregular menstrual bleeding had highly enriched *Gemminger* sp900540595 (*q* < 0.0001) and depleted *Stoquefichus massiliense* (*q* < 0.0001)*, Lachnospiraceae CAG-81 sp009917545* (*q* < 0.0001)*, Atopobiaceae UBA1367 sp902779675* (*q* < 0.0001)*, Parvimonas sp000223315* (*q* < 0.0001)*, Atopobium deltae* (*q* < 0.0001)*,* and *Buttiauxella agrestis A* (*q* < 0.0001) (Additional file 1: Fig. S24, Additional file 3: Table S19)*.*

To identify if it was the condition or the presence of blood in vaginal samples altering the microenvironment, we performed a sub-analysis among those with blood absent or present in vaginal samples. This did not yield similar results to the differential vaginal bacteria identified in AUB, heavy, and irregular menstrual bleeding, suggesting the observed alterations were due to the AUB (Additional file 1: Fig. S25 and S26, Additional file 2: Table S10).

### Immune profiles of co-occurring conditions

To identify relationships between the immune proteins and co-occurring conditions of CPP, we analyzed the levels of immune markers compared to diagnosed AUB, fibroids, and ovarian cysts. Diagnosis of ovarian cyst revealed no altered immune markers except prior to FDR adjustment, where PDGF-AB/BB (*p* = 0.044) levels were elevated (Fig. 6C, D, Additional file 4: Table S27). The analysis also revealed a decrease in IL-1Ra (*q* = 0.024, *q* = 0.035) in AUB and fibroids compared to no AUB or fibroids (Fig. 6C, D, Additional file 4: Tables S28 and S29). Patients with fibroids had uniquely decreased levels of IL-17A (*q* = 0.023), EGF (*q* = 0.034), and an elevation of macrophage inflammatory protein-1 beta (MIP-1β) (*q* = 0.040) (Fig. 6C, D, Additional file 4: Tables S28 and S29). Among patients with AUB, those with heavy menstruation had lower levels of IP-10 (*q* = 0.022) (Additional file 1: Fig. S27), while those with irregular menstruation overall had lower levels of immune markers IL-1Ra (*q* = 0.002) and IL-17A (*q* = 0.002) (Additional file 1: Fig. S27).

To identify the impact of blood in the CVL samples, we analyzed the significantly altered markers in samples observed with and without blood. Alteration of 22/41 protein biomarkers between CVLs with and without blood. RANTES (*q* < 0.00001), IL-6 (*q* < 0.00001), TNF⍺ (*q* < 0.00001), MCP-1 (*q* = 0.0003), FGF-2 (*q* = 0.001), platelet-derived growth factor AA/BB (PDGF-AB/BB) (*q* = 0.002), MIP-1beta (*q* = 0.003), and IL-10 (*q* = 0.009) were all increased (Additional file 1: Fig. S27, Additional file 4: Table S30). IL-5 (*q* < 0.001), IL-12p40 (*q* < 0.00001), IL-15 (*q* < 0.00001), IFN (*q* < 0.00001), IL-2 (*q* < 0.00001), Flt-3L (*q* < 0.00001), IL-7 (*q* < 0.00001), IL-9 (*q* < 0.00001), IL-17A (*q* < 0.00001), IL-13 (*q* < 0.00001), IL-3 (*q* < 0.00001), TNFβ (*q* = 0.004), IP-10 (*q* = 0.03), and IL-1⍺ (*q* = 0.049) were all decreased (Additional file 1: Fig. S27, Additional file 4: Table S30).

### Microbiome and immune marker interplay in benign gynecologic conditions

To understand the interactions between vaginal immune proteins and enriched or depleted vaginal bacteria of chronic pelvic pain and co-occurring gynecologic conditions, we performed Spearman correlation analysis supervised by immune marker type: cytokines, chemokines, growth factors, and immune checkpoints.

**Fig. 7:**
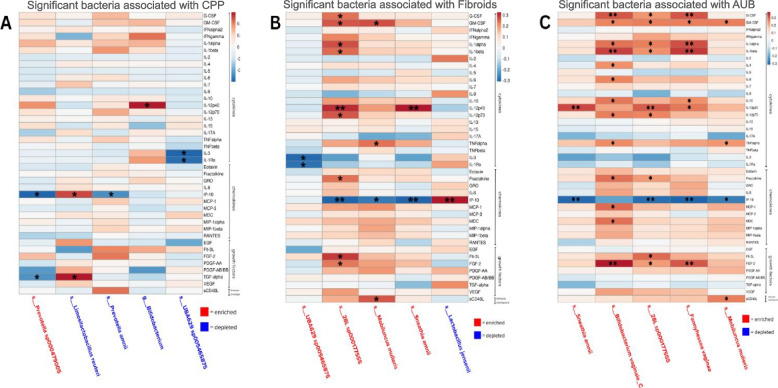
Correlations of differentially abundant microbes and cervicovaginal immune markers. A correlation analysis between cervicovaginal lavage levels of 41 immune proteins with significant differentially abundant vaginal taxa of subjects from analysis groups: **A** chronic pelvic pain (CPP), **B** fibroids, **C** abnormal uterine bleeding (AUB). Correlation coefficients (*r*) were calculated using Spearman’s rank correlation, where positive (red) and negative (blue) correlations are depicted as a heatmap. *p*-values < 0.05 were significant, where * is denoted as < 0.05, ** is denoted as < 0.01, *** < 0.001, and **** < 0.0001 *p*-value. Taxa labels in red signify that a bacterial species was enriched in the samples, and taxa in blue signifies that a bacterial species was depleted in the samples from each analysis group

Correlation analysis between immune proteins and vaginal bacteria significantly altered in patients with CPP revealed five immune proteins with significant correlations (Fig. 7A). Correlation analysis between immune proteins and vaginal bacteria altered in patients with co-occurring conditions such as AUB and fibroids revealed correlations of 14 and 17 immune proteins, respectively (Fig. 7B, C). *Lachnospiraceae UBA629*A, which was depleted in patients with CPP and enriched in patients with fibroids, was negatively correlated to IL-3 (*p* < 0.05) and IL-1Ra (*p* < 0.05). IL-1Ra was also upregulated in patients with CPP and no endometriosis. *Prevotella amnii* (*p* < 0.05), depleted in patients with CPP, and *Prevotella* sp000479005 (*p* < 0.05), enriched in CPP, were negatively correlated to interferon-gamma-induced protein-10 (IP-10). *Limosilactobacillus reuteri* (*p* < 0.05), depleted in CPP, and *Limosilactobacillus coleohominis* (*p* < 0.01), depleted in blood in the sample, were positively correlated with IP-10, which has antiangiogenic properties in a study of endometriosis [58]. *L. jensenii* (*p* < 0.01) was only depleted in fibroids and, like *L. reuteri,* had a positive correlation to IP-10, while *Sneathia amnii* (*p* < 0.01), *Mobiluncus mulieris* (*p* < 0.05), and *Megasphaera lornae* (*p* < 0.01) were enriched in fibroids and AUB and negatively correlated. *Fannyhessea vaginae* (*p* < 0.01) was enriched in AUB only but showed the same trend. *L. reuteri* (*p* < 0.05) was also positively correlated to TGFα, while *Prevotella* sp000479005 (*p* < 0.05) was negatively correlated. IL-12 p40, a proinflammatory interleukin, was positively correlated with an unclassified *Bifidobacterium* species (*p* < 0.05) that was enriched in controls and depleted in those with blood in the sample (Fig. 7A and Additional file 1: Fig. S28). However, IL-12 p40 was also positively correlated to *S. amnii* and *M. lornae,* which were enriched in AUB and fibroids. The motile bacterium, *M. mulieris,* was enriched in AUB and was the only bacteria to positively correlate to the immune checkpoint marker CD40 (*p* < 0.05). Soluble CD40 ligand in the uterus is mostly sourced by platelets [59], which could link to changes in the host environment favoring pathogenic microbes. Proinflammatory cytokine TNFα was positively correlated with *M. mulieris* enriched in fibroids and AUB (*p* < 0.05) and *Bifidobacterium vaginale C* enriched in AUB (*p* < 0.05). GM-CSF, promoter of proliferation and migration on endometrial cells [60], was positively correlated with *M. mulieris* (*p* < 0.05), *M. lornae* (*p* < 0.05), *B. vaginale C* (*p* < 0.05), and *F. vaginae* (*p* < 0.05). G-CSF, associated with angiogensis [61], was positively correlated to *M. lornae* (*p* < 0.05), *B. vaginale C* (*p* < 0.01), and *F. vaginae* (*p* < 0.01). These bacteria were also positively correlated to proinflammatory markers IL-1α (*p* < 0.05, *p* < 0.05, *p* < 0.01, respectively) and IL-1β (*p* < 0.05, *p* < 0.01, *p* < 0.01, respectively). Additionally, these bacteria were positively correlated to fibroblast growth factor 2 and FGF-2 (*p* < 0.05, *p* < 0.01, *p* < 0.01, respectively), which has been associated with proliferation, migration, and invasion of ectopic stromal cells [62]. *M. lornae* and *B. vaginale C* were also positively correlated with IL-12 p70 (*p* < 0.05, *p* < 0.05, respectively) and fractalkine (*p* < 0.05, *p* < 0.05, respectively). *B. vaginale C* was only enriched in AUB but was the only bacteria to have significantly positive correlations to MCP-1 (*p* < 0.05), MDC (*p* < 0.05), IL-4 (*p* < 0.05), and IL-6 (*p* < 0.05). Overall, this analysis revealed correlations between microbiota and immune proteins associated with CPP or other co-occurring gynecologic conditions. This demonstrates the underappreciated potential of bacterial contribution to the microenvironment of co-occurring conditions.

## Discussion

**Fig. 8:**
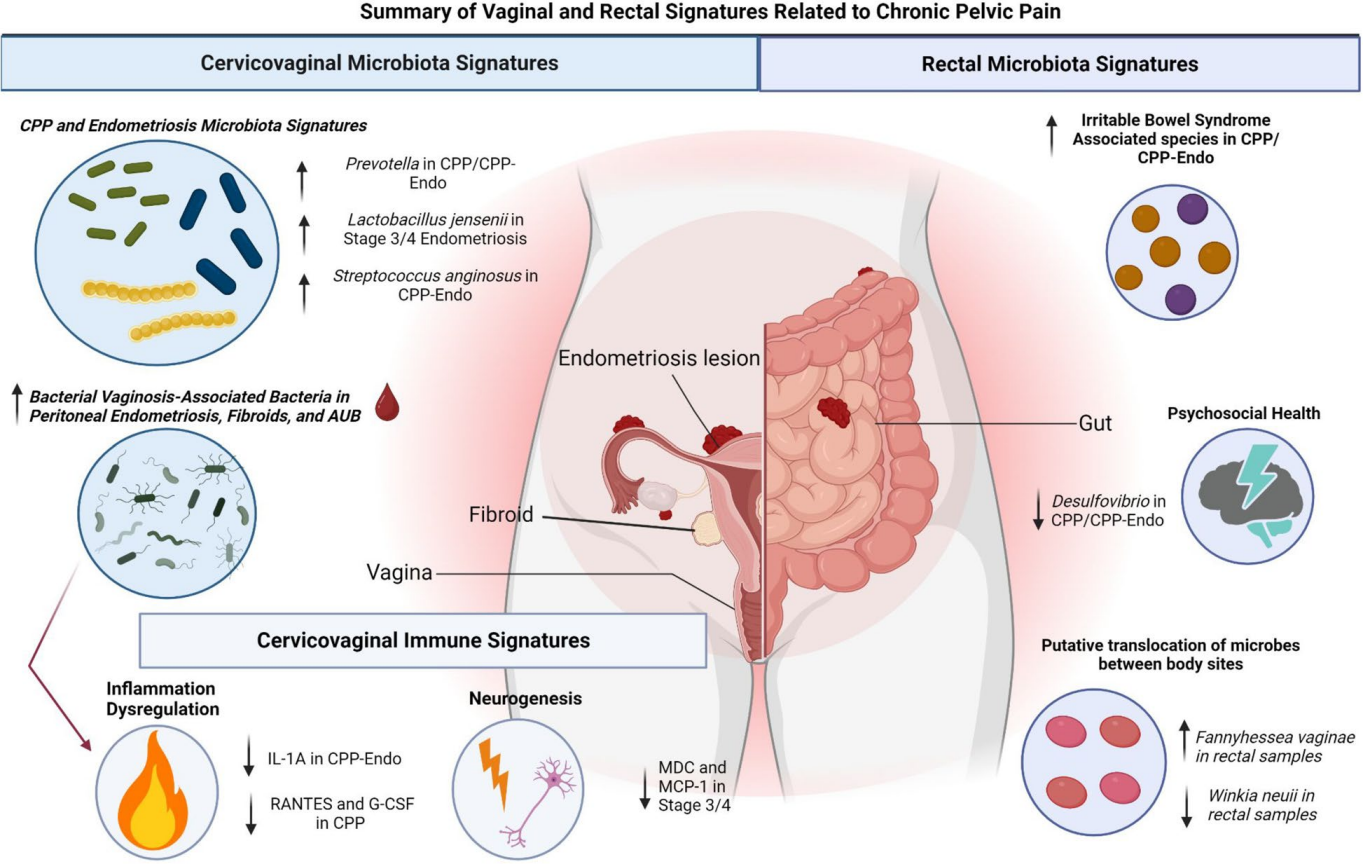
Summary of findings of microbiome and cervicovaginal immune markers for CPP and CPP with endometriosis. Dysbiotic vaginal and rectal bacteria might affect the etiology of chronic pelvic pain with and without endometriosis. An increase in bacterial-vaginosis-associated bacteria such as *Megasphaera*, *Lachnospiraceae*, *Fannyhessea*, *Mobiluncus*, *Bifidobacterium/Gardnerella* were associated with peritoneal endometriosis, fibroids, and abnormal uterine bleeding. These microorganisms also revealed a positive correlation with proinflammatory immune mediators and growth factors. Our cervicovaginal lavage samples also revealed a decrease in IL-1a in CPP-Endo and RANTES and G-CSF in CPP, revealing a dysregulation in inflammatory pathways for chronic pelvic pain with or without endometriosis. Decreased MDC and MCP-1 were also observed in endometriosis stage 3/4, which may link to neurogenesis, which is observed more in endometriosis stage 1/2. This linkage may also interplay with the increase of *Lactobacillus jensenii* observed in endometriosis stage 3/4. Increased *Prevotella* was observed in both CPP and CPP-Endo, while *Streptococcus anginosus* was more abundant in CPP-Endo than CPP. Our rectal samples revealed an increase in bacteria previously associated with rectal dysbiosis in colitis and irritable bowel disorder, which has also been associated with chronic pelvic pain; some of these bacteria, including Stoquefichus and Ruminococcus that were increased in both CPP and CPP-Endo. A shared depletion of Desulfovibrio was also observed in CPP and CPP-Endo. Finally, a sharing of vaginal taxa such as *Fannyhessea* and *Winkia* in the rectal samples was observed, which has previously been linked to systemic dysbiosis and sharing of microbes between sites

Nonspecific symptoms, such as chronic pelvic pain and delays in diagnosis, make understanding the underlying etiology of endometriosis challenging [63]. Additionally, since the clinical severity of endometriosis symptoms does not correlate with the surgical stage [64], investigating the relationship of biological readouts (immune markers and microbiota) may reveal mechanistic insights into the disease process and provide targets for specified treatment for types of CPP. We aimed to identify key markers to distinguish chronic pelvic pain with and without endometriosis utilizing the vaginal and rectal microbiota as well as soluble immune markers in cervicovaginal lavages (Fig. 8).

No single microbe or immune marker has yet been validated for specific diagnosis of endometriosis in the USA, but some have been investigated in other countries such as France [4, 65]. IL-1α has been identified as a potential marker for endometriosis [66], and we observed that it was increased in patients with CPP with and without endometriosis. TNF-α in endometriosis has also been previously studied [67], but there has been insufficient evidence that anti-TNFα drugs are effective in treating pain symptoms in patients with endometriosis [68]. Our pilot study found that VEGF was decreased in CPP and CPP-Endo compared to controls. VEGF has been associated with angiogenesis in endometriosis, and anti-VEGF treatment can inhibit the growth of endometriosis in animal models [69]. Our findings may differ from other studies as patients in our control group also describe heavy menstrual or irregular bleeding, meaning that abnormal bleeding may be more related to the VEGF signature observed in benign gynecologic conditions. MCP-1 can be expressed by ectopic endometrial implants [70] and stimulates cell attachment [71] and angiogenesis [72], while MDC is involved in dendritic cell and T-helper cell recruitment [73] and neurogenesis that may be involved in pain [74]. Both were associated with stage 1/2 in our study, and MDC was also associated with endometriosis of the ovary. RANTES was the only immune factor that was enriched in patients with peritoneal endometriosis; this immune mediator has been observed to be upregulated in stromal cells stimulated by a proinflammatory environment [75]. In a murine model, RANTES antagonist attenuated inflammation and pain [76]. IL-1Ra was increased in CPP and decreased in patients with fibroids and AUB; this anti-inflammatory marker has a protective effect against pain hypersensitivity [77], which may be why it is overproduced in a pelvic pain environment outside of endometriosis diagnosis.

With regard to microbiome findings, vaginal *L. jensenii* was increased in the CPP-Endo group, which differs from other reports. One study previously observed a statistically significant increase of *Clostridium disporicum*, as well a decrease in several *Lactobacillus* species (*L. jensenii, L. reuteri, L. iners*) in the vaginal microbiome of patients with CPP-Endo [13]. CA-125 values were used along with the relative abundance of *C. disporicum and L. reuteri* as a diagnostic tool to differentiate from patients with CPP and no endometriosis, reporting a sensitivity of 89.19% but a specificity of 52.0% [27]. Other studies have found vaginal *L. jensenii* to be decreased in patients with CPP-Endo or increased in early-stage endometriosis [28]. Therefore, further research is needed to conclude the association of vaginal *Lactobacillus* with the presence, location, and stage of endometriosis and other factors that may confound these studies. The pathobiont *Streptococcus* has been associated with endometriosis in multiple studies, without the identification of species [78, 79]. We identified *Streptococcus anginosus,* a highly inflammatory species of Streptococcus [80]*,* associated with the vaginal microbiome of patients with CPP-Endo compared to CPP alone. *Streptococcus* at the genus level has been previously associated with endometriosis [78, 79]. However, species-level identification of *S. anginosus* has not yet been observed. *Prevotella* has been associated with endometriosis in prior research, with some studies reporting an increase in cervical/vaginal samples and others reporting a decrease [28]. We observed an increased vaginal abundance of *Prevotella sp000479005* in CPP despite endometriosis diagnosis and decreased vaginal abundance of *Prevotella amnii* in CPP without endometriosis. This apparent discrepancy may be due to overlapping with other co-occurring gynecologic conditions of CPP or may be due to species-specific changes [81]. Our study identified that fibroids and abnormal uterine bleeding, including heavy and irregular menstrual bleeding, were associated with key bacterial vaginosis-associated bacteria such *Bifidobacterium vaginale C*, *Megasphaera lornae*, *Anaerococcus vaginalis, Fannyhessea vaginae*, *Sneathia amnii*, and *Mobiluncus mulieris*. Importantly, these bacteria did not overlap with microbes observed in samples containing blood. This suggests the microbiome may offer a glimpse into the microbial structure of different conditional environments that overlap with chronic pelvic pain or endometriosis and may be an additional feature to investigate further to understand the symptomology of CPP and endometriosis. These findings are strengthened as continued significance is observed despite the presence of blood at the time of collection.

A human study suggested that microbiome alterations may differ depending on the stage or location of endometriosis [82]. Changes in the microbiome that align with the location of endometriosis could provide insights to better understand the role microbiota play in the severity of endometriosis and the relationships of symptoms such as chronic pelvic pain. *Anaerococcus* was noted to correlate with stage 3/4 disease [82]. Here, however, we found *Anaerococcus*, along with other species, to be associated with stage 1/2 endometriosis. Further, *Winkia neuii* was depleted in the rectum and enriched in the vagina for stage 3⁄4 endometriosis. Others have reported the presence of enriched vaginal taxa in the rectum [79]. This may signal the transmission of microbes between body sites, which has previously been suggested to be a signature of gynecologic disease [83]. Additionally, we found vaginal microbiome alterations based on the location of endometriosis, with an increase in *Streptococcus* sp., *L. crispatus*, and *Prevotella* in ovarian endometriosis. *Prevotella* and *Streptococcus* have been associated with pelvic inflammatory disease and can be found in tissues of the upper reproductive tract [84]. Further, a higher abundance of *Sneathia amnii, Megasphaera lornae, Fannyhessea vaginae,* and *Escherichia* was observed in peritoneal endometriosis. *Fannyhessea vaginae* was enriched in both the rectal and vaginal microbiome of peritoneum endometriosis. These BV-associated bacteria have also been associated with increased inflammation in the lower and upper reproductive tract [85, 86]. It is possible that ascending microbiota from the lower reproductive tract could impact inflammation and lesion progression at the differing sites of endometriosis, and this inflammation can induce pain [87, 88]. However, future studies would be needed to advance this theory, including tissue samples of the upper female reproductive tract and intraperitoneal area. Recent links have been between the rectal pathogen *Fusobacterium nucleutum* and lesion growth [54]. The vaginal *Fusobacterium nucleus* was highly enriched in patients with endometriosis in multiple locations, and *Fusobacterium animalis* was associated with irregular menstrual bleeding and CPP alone when compared to CPP-Endo. Endometriosis at multiple locations had the most microbiome differences, which may further support the rectal microbiome’s link to systemic modulation via inflammation or hormones for gynecologic conditions such as endometriosis. This supports the hypothesis that the alterations in the microbiome may be associated with endometriosis disease severity and since the rASRM staging system is a composite of both location and extent of disease, this may explain the differences observed between studies.

Previous studies identified that a mouse model of induced endometriosis led to a shift in fecal flora, and patients with endometriosis have alterations in gut microbiota compared to healthy controls [89, 90]. Our study observed a decrease in *Chryseobacterium* in CPP alone; this bacteria has been associated with unsuccessful pregnancy outcomes [91] and degradation of collagen and keratin [92], which might be important for other gynecologic diseases outside of CPP. We also observed an increase in *Stoquefichus massiliensis*, *Ruminococcus E*, *Anaerostipes*, *Lacrimispora,* and *Bulleidia* in the rectal samples of patients from both CPP groups. *Anaerosacchariphilus* sp002160765 was also depleted in CPP-Endo when compared to CPP alone. This species is an aldehyde producer, which may trigger oxidative stress and neurogenic inflammation in the gut [93, 94]. Further, endometriosis is highly correlated to gastrointestinal diseases such as irritable bowel syndrome (IBS) and, potentially, inflammatory bowel disease (IBD) [95, 96]. *Ruminococcus* and *Stoquefichus* increased in CPP have also been associated with IBS or colitis [95, 97–99]. Although many patients in our study did not report a previous history of IBS or IBD, future studies into gastrointestinal symptoms in patients with CPP and endometriosis may provide insight into how the gut microbiome may relate to the etiology of abdominal pain.

We identified increased abundance of *Prevotella*, *Parasporobacterium paucivorans*, *Peptoniphilaceae KA00134*, *Buttiauxella agrestis A*, *Agathobacter* sp., *Prevotella colorans*, *Bifidobacterium kashiwanohense A*, *Ruminococcus_E*, and *Akkermansia* sp*.* in stage 3/4 endometriosis. Investigating the role of these microbes may help identify microbes that either solely benefit from an estrogen-dominant environment or those that might directly induce abdominal discomfort or pain. Given that probiotic treatment has shown benefits in other conditions associated with dysbiosis, such as IBS [100], there may also be a role for supplementation in the treatment of CPP. Although the optimal regimen is unknown, oral *Lactobacillus* has shown some benefit in decreasing dysmenorrhea scores in one small pilot study [101], and *Lactobacillus* spp*.* have also been used with *Bifidobacterium longum* to alleviate visceral hypersensitivity [102]. Including patient-reported diet, probiotic, or herbal medicine usage could be a future direction of research that investigates microbial modulation for symptom management. Conversely, the presence of pathogenic species presents an opportunity to study antibiotic treatments, which have been shown to decrease lesion size and inflammation in animal models of endometriosis [103].

Strengths of this study include our strict definition of comparison groups, requiring pathological confirmation of endometriosis, which allowed us to specifically evaluate the microbiome and cervicovaginal immune profile of patients with non-endometriosis-related chronic pelvic pain, as well as those with endometriosis. Additionally, this study evaluated patients with co-occurring gynecologic conditions, which were more prevalent in patients with endometriosis, highlighting the complex conditions that can make clinical treatment and study of pelvic pain challenging. Also, the collection procedure used was standardized and performed at a single institution, then samples were processed and analyzed in a lab experienced in microbiome analysis.

Limitations of our pilot study include the sample size, particularly in the control and CPP groups, which resulted from our patient population and the disruption in elective surgeries during the global COVID-19 pandemic. This study broadly explored individuals seeking treatment for chronic pelvic pain. However, there was a lack of data regarding the specific type and severity of pain, which could serve as valuable additional information for future studies examining the relationship between the microbiome and pelvic pain. Also, the presence of co-occurring gynecologic conditions such as AUB and fibroids within our groups, including the control group, contributed to alterations in the microbiome, as discussed, and may result in misclassification. The focus of our study was not adenomyosis, but in the future, it may warrant focusing on patients' diagnoses with co-occurring conditions of CPP, including adenomyosis. The presence of blood likely affects the concentration of immune protein markers in our samples but as blood was present in a minority of samples (34%) and evenly distributed between diagnosis groups, observed differences should remain valid, and we would expect the presence of blood to attenuate any associations.

## Conclusions

We identified changes in the microbiome and local immune markers that were associated with CPP and differentiated CPP with and without endometriosis compared to women without chronic pelvic pain or endometriosis. Identification of microbial and immune differences based on site and stage of endometriosis suggests the microenvironment of peritoneal lesions or early disease may differ from other manifestations of endometriosis. A sub-analysis of our samples revealed that AUB and fibroids were associated with BVAB, suggesting that co-occurring gynecologic conditions contribute to microbiome changes and symptoms in patients with CPP. This study provides foundational knowledge on the etiology of CPP with and without endometriosis and co-occurring gynecologic conditions and provides targets for validation in larger cohorts.

### Supplementary Information

Additional file 1: Figure S1: Post-operative co-occurring conditions amongst disease groups of chronic pelvic pain, chronic pelvic pain with endometriosis, and surgical controls. Figure S2: Vaginal Lactobacillus abundances across diagnosis groups. Figure S3: Microbial diversity of patients diagnosed with chronic pelvic pain, chronic pelvic pain with endometriosis, and surgical controls. Figure S4: Differentially abundant taxa of CPP, CPP-Endo compared to surgical controls. Figure S5: Differentially abundant taxa of CPP-Endo compared to CPP. Figure S6: Cluster metadata analysis from hierarchical heatmap of immune data. Figure S7: Cytokine differences between hierarchical heatmap for cluster 1 and cluster 2. Figure S8: Microbial alpha-diversity of patients diagnosed with endometriosis-by-endometriosis stage and location in vaginal and rectal samples. Figure S9: Microbial beta-diversity of patients diagnosed with endometriosis-by-endometriosis stage and location in vaginal and rectal samples. Figure S10: Vaginal *Lactobacillus* abundances across endometriosis stage and location. Figure S11: Differentially abundant taxa of endometriosis stage 3/4 compared to endometriosis stage 1/2. Figure S12: Differentially abundant taxa of endometriosis location sites. Figure S13: Microbial alpha-diversity of patients diagnosed with abnormal uterine bleeding, fibroids, and ovarian cysts in vaginal samples. Figure S14: Microbial alpha-diversity in rectal samples of patients diagnosed with abnormal uterine bleeding, fibroids, and ovarian cysts. Figure S15: Microbial beta-diversity of patients diagnosed with abnormal uterine bleeding, fibroids, and ovarian cysts in vaginal and rectal samples. Figure S16: Vaginal *Lactobacillus* abundances across co-occurring gynecologic conditions. Figure S17: HeatTree identifies differentially abundant taxa amongst co-occurring conditions: abnormal uterine bleeding, fibroids, and ovarian cysts in vaginal samples. Figure S18: HeatTree identifies differentially abundant taxa amongst co-occurring conditions: abnormal uterine bleeding, fibroids, and ovarian cysts in rectal samples. Figure S19: Vaginal taxa profiles of co-occurring condition: abnormal uterine bleeding, fibroids, and ovarian cysts in CPP cohort. Figure S20: Rectal taxa profiles of co-occurring condition: abnormal uterine bleeding, fibroids, and ovarian cysts in CPP cohort. Figure S21: Microbial diversity of patients diagnosed with heavy menstrual bleeding and irregular menstrual bleeding in vaginal and rectal samples. Figure S22: Vaginal *Lactobacillus* abundances across menstrual bleeding symptoms. Figure S23: Differentially abundant taxa of heavy menstrual bleeding. Figure S24: Differentially abundant taxa of irregular menstrual bleeding. Figure S25: Microbial diversity of blood present in vaginal samples. Figure S26: Differentially abundant taxa of blood observed in the vaginal sample. Figure S27: Immune Protein concentrations comparisons of heavy and irregular menstrual bleeding and blood within the sample. Figure S28: Heatmap of differentially abundant taxa of blood observed in vaginal samples and correlation to immune markers.

Additional file 2: Table S1: Vaginal differential abundance statistical results of the groups’ CPP and CPP-Endo compared to surgical controls. Table S2: Vaginal differential abundance statistical results of the groups’ CPP-Endo compared to CPP. Table S3: Vaginal differential abundance statistical results of the groups’ endometriosis stage 3/4 compared to endometriosis stage 1/2. Table S4: Vaginal differential abundance statistical results of endometriosis locations peritoneum, multiple locations, or other sites compared to endometriosis of the ovary. Table S5: Vaginal differential abundance statistical results of the groups’ ovarian cysts compared to no ovarian cysts. Table S6: Vaginal differential abundance statistical results of the groups’ fibroids compared to no fibroids. Table S7: Vaginal differential abundance statistical results of the groups’ abnormal uterine bleeding compared to no abnormal uterine bleeding. Table S8: Vaginal differential abundance statistical results of the groups’ heavy menstrual bleeding compared to no heavy menstrual bleeding. Table S9: Vaginal differential abundance statistical results of the groups’ irregular menstrual bleeding compared to no irregular menstrual bleeding. Table S10: Vaginal differential abundance statistical results of the groups’ blood in the sample compared to no blood in the sample.

Additional file 3: Table S11: Rectal differential abundance statistical results of the groups’ CPP and CPP-Endo compared to surgical controls. Table S12: Rectal differential abundance statistical results of the groups’ CPP-Endo compared to CPP. Table S13: Rectal differential abundance statistical results of the groups’ endometriosis stage 3/4 compared to endometriosis stage 1/2. Table S14: Rectal differential abundance statistical results of endometriosis locations peritoneum, multiple locations, or other sites compared to endometriosis of the ovary. Table S15: Rectal differential abundance statistical results of the groups’ ovarian cysts compared to no ovarian cysts. Table S16: Rectal differential abundance statistical results of the groups’ fibroids compared to no fibroids. Table S17: Rectal differential abundance statistical results of the groups’ abnormal uterine bleeding compared to no abnormal uterine bleeding. Table S18: Rectal differential abundance statistical results of the groups’ heavy menstrual bleeding compared to no heavy menstrual bleeding. Table S19: Rectal differential abundance statistical results of the groups’ irregular menstrual bleeding compared to no irregular menstrual bleeding.

Additional file 4: Table S20: immunoproteomic statistical results of the groups’ CPP compared to surgical controls. Table S21: immunoproteomic statistical results of the groups’ CPP-Endo compared to surgical controls. Table S22: immunoproteomic statistical results of the groups’ CPP compared to CPP-Endo. Table S23: immunoproteomic statistical results of the groups’ endometriosis stage 3/4 compared to stage 1/2. Table S24: immunoproteomic statistical results of the groups’ other endometriosis locations compared to endometriosis of the ovary. Table S25: immunoproteomic statistical results of the group endometriosis on peritoneum compared to endometriosis of the ovary. Table S26: immunoproteomic statistical results of the groups’ endometriosis at multiple locations compared to endometriosis on the ovary. Table S27: immunoproteomic statistical results of the groups’ ovarian cysts compared to no ovarian cysts. Table S28: immunoproteomic statistical results of the groups’ fibroids compared to no fibroids. Table S29: immunoproteomic statistical results of the groups’ abnormal uterine bleeding compared to no abnormal uterine bleeding. Table S30: immunoproteomic statistical results of the groups’ blood in the sample compared to no blood in the sample.

## Data Availability

The data that support this study’s findings are available in the paper and additional files of supplemental figures and statistical results. 16S rRNA gene sequences were deposited in the National Center for Biotechnology Information (NCBI) and a link to the data is provided at https://www.ncbi.nlm.nih.gov/bioproject/PRJNA1071129.

## Abbreviations

AUB: Abnormal uterine bleeding
BV: Bacterial vaginosis
BVAB: Bacterial vaginosis-associated bacteria
CVL: Cervicovaginal lavage
Endo: Endometriosis
IBS: Irritable bowel syndrome
