# Supplementary material for: Vaginal and rectal microbiome contribute to genital inflammation in chronic pelvic pain
Source: BMC Med. 2024 Jul 8;22:283. doi: 10.1186/s12916-024-03500-1 (PMC11229265; doi:10.1186/s12916-024-03500-1)
Supplement: Supplementary file 1 — Additional file 1: Figure S1: Post-operative co-occurring conditions amongst disease groups of chronic pelvic pain, chronic pelvic pain with endometriosis, and surgical controls. Figure S2: Vaginal Lactobacillus abundances across diagnosis groups. Figure S3: Microbial diversity of patients diagnosed with chronic pelvic pain, chronic pelvic pain with endometriosis, and surgical controls. Figure S4: Differentially abundant taxa of CPP, CPP-Endo compared to surgical controls. Figure S5: Differentially abundant taxa of CPP-Endo compared to CPP. Figure S6: Cluster metadata analysis from hierarchical heatmap of immune data. Figure S7: Cytokine differences between hierarchical heatmap for cluster 1 and cluster 2. Figure S8: Microbial alpha-diversity of patients diagnosed with endometriosis-by-endometriosis stage and location in vaginal and rectal samples. Figure S9: Microbial beta-diversity of patients diagnosed with endometriosis-by-endometriosis stage and location in vaginal and rectal samples. Figure S10: Vaginal Lactobacillus abundances across endometriosis stage and location. Figure S11: Differentially abundant taxa of endometriosis stage 3/4 compared to endometriosis stage 1/2. Figure S12: Differentially abundant taxa of endometriosis location sites. Figure S13: Microbial alpha-diversity of patients diagnosed with abnormal uterine bleeding, fibroids, and ovarian cysts in vaginal samples. Figure S14: Microbial alpha-diversity in rectal samples of patients diagnosed with abnormal uterine bleeding, fibroids, and ovarian cysts. Figure S15: Microbial beta-diversity of patients diagnosed with abnormal uterine bleeding, fibroids, and ovarian cysts in vaginal and rectal samples. Figure S16: Vaginal Lactobacillus abundances across co-occurring gynecologic conditions. Figure S17: HeatTree identifies differentially abundant taxa amongst co-occurring conditions: abnormal uterine bleeding, fibroids, and ovarian cysts in vaginal samples. Figure S18: HeatTree identifies differentia [file 12916_2024_3500_MOESM1_ESM.docx]

**
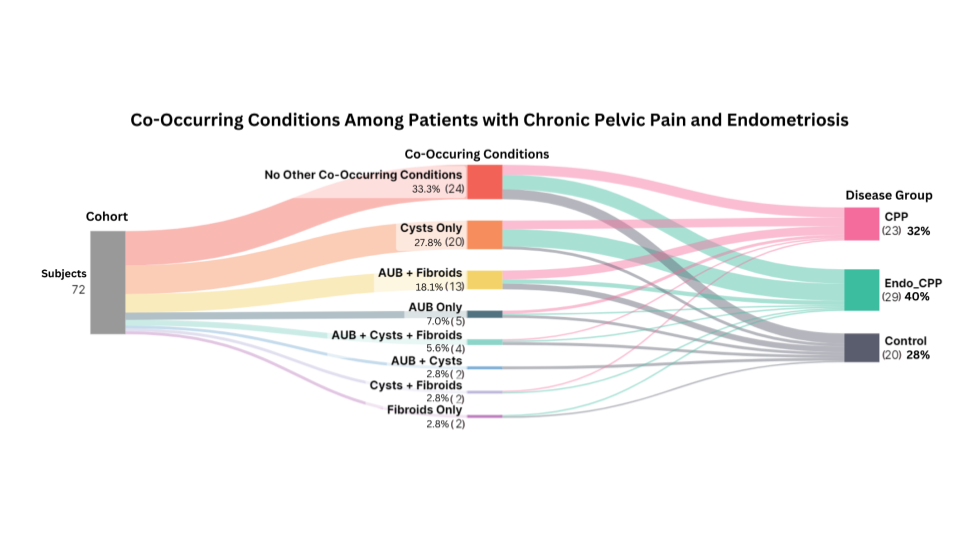
Figure S1: Post-operative co-occurring conditions amongst disease groups of chronic pelvic pain, chronic pelvic pain with endometriosis, and surgical controls.** The Sankey network showcases the complexity of the cohort in regard to co-occurring conditions and disease groups. The cohort (n=72) is comprised of 72 subjects, 48 of whom had co-occurring conditions other than endometriosis. On the right side of the Sankey, the disease groups identified in the study are pink, representing patients in the disease group with chronic pelvic pain. Green represents patients in the disease group with chronic pelvic pain with endometriosis, and dark grey represents patients in the control group. In the middle section of the Sankey diagram, other co-occurring conditions are shown: red represents no co-occurring conditions with 24 subjects, orange represents those with cysts as the only other occurring condition with 20 subjects, yellow represents those with abnormal uterine bleeding as occurring condition for 13 subjects, dark green representing those with abnormal uterine bleeding as the only other occurring condition for five subjects, mint green representing those with abnormal uterine bleeding, cysts, and fibroids as the only occurring condition for four subjects, blue representing those with abnormal uterine bleeding and cysts as the only occurring condition for two subjects, light purple representing those with cysts and fibroids as the only occurring condition for two subjects, and purple representing those with fibroids as the only occurring condition for two subjects.


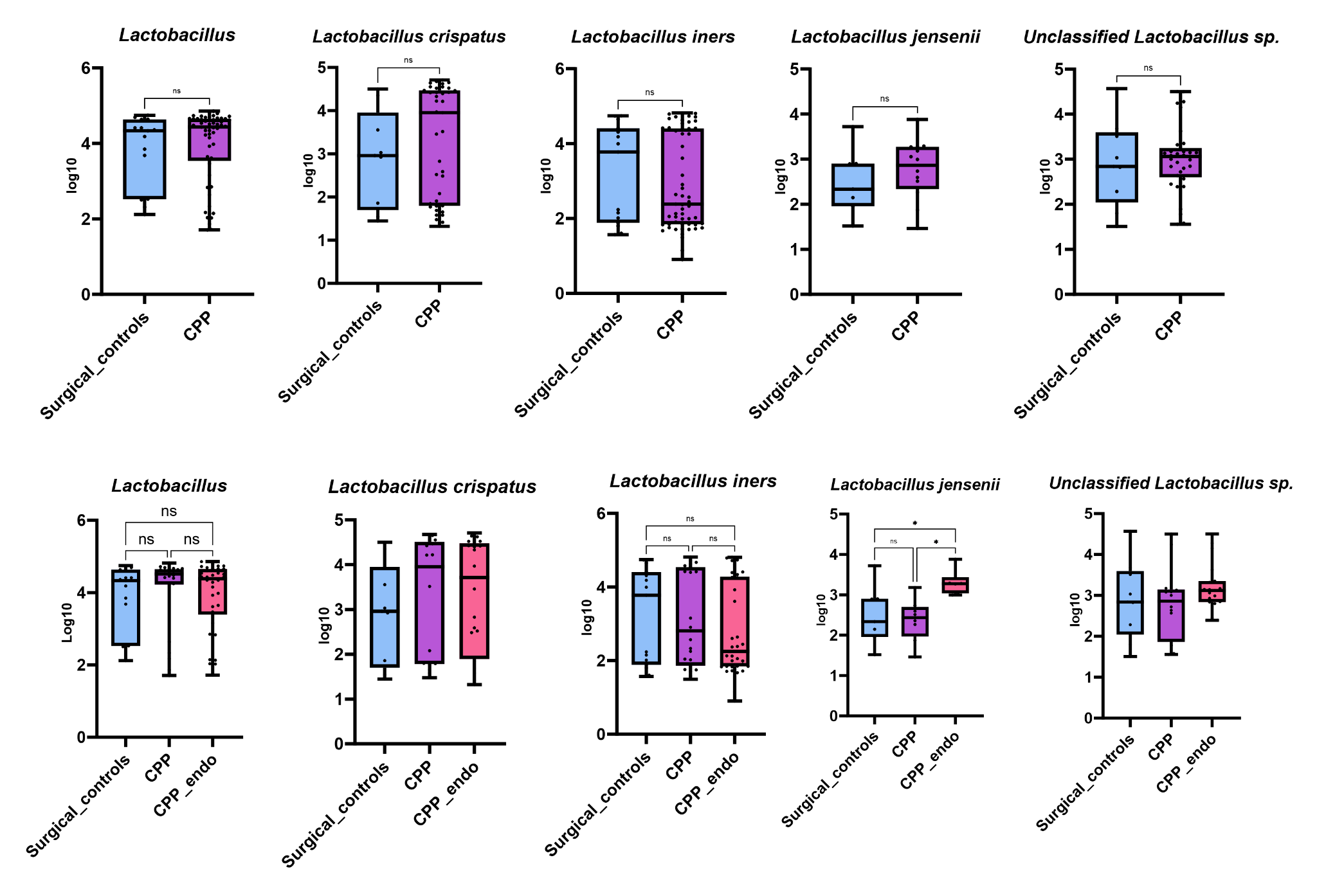
**Figure S2: Vaginal *Lactobacillus* abundances across diagnosis groups.** Abundance plots of Lactobacillus genus and species amongst disease groups. **(A)** Dot plots of Lactobacillus genus and species log10 transformed amongst patients diagnosed with chronic pelvic pain (purple) and surgical controls (light blue). **(B)** Dot plots of Lactobacillus genus and species log10 transformed amongst patients diagnosed with chronic pelvic pain without endometriosis (pink), chronic pelvic pain with endometriosis (purple), and surgical controls (light blue). Additional abundance difference testing is performed by the Mann-Whitney test where “ns” is not significant, * is <0.05,  ** is <0.01, *** <0.001, and **** <0.0001 p-value.


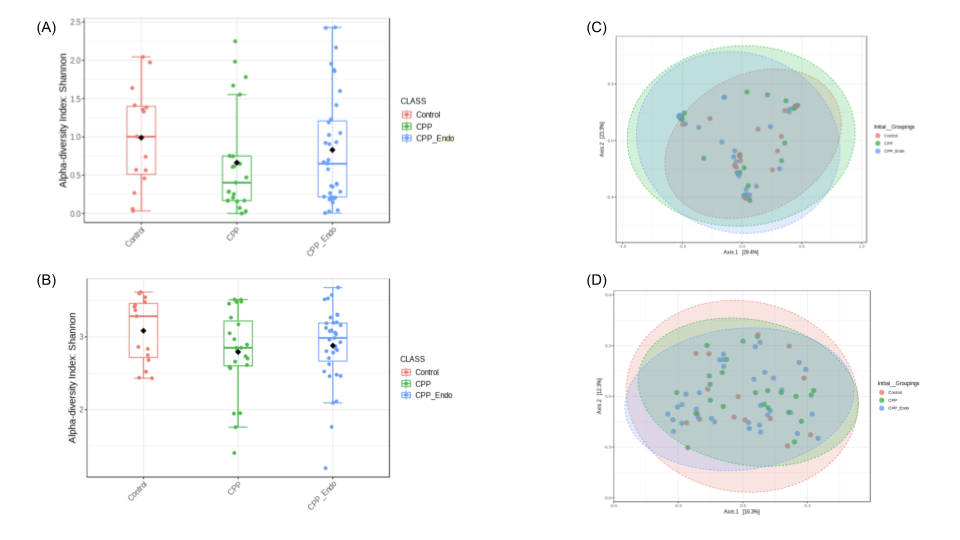


**Figure S3: Microbial diversity of patients diagnosed with chronic pelvic pain, chronic pelvic pain with endometriosis, and surgical controls.**Boxplots of alpha-diversity differences between women with (green) chronic pelvic pain (CPP), (blue) CPP and endometriosis (endo), (red) and surgical controls (controls). Vaginal samples **(A)** at the species level utilizing the Shannon index (p-value: 0.37112; [ANOVA] F-value: 1.0054); rectal samples **(B)** at the species level utilizing the Shannon index (p-value: 0.24934; [ANOVA] F-value: 1.4169). A beta-diversity measure demonstrates the dissimilarity of taxa at the species level in women with (green) CPP, (blue) CPP with Endo (red), and controls. Vaginal samples **(C)** ([PERMANOVA] F-value: 1.125; R-squared: 0.031141; p-value: 0.358); **(D)** rectal samples ([PERMANOVA] F-value: 0.73729; R-squared: 0.020631; p-value: 0.868).


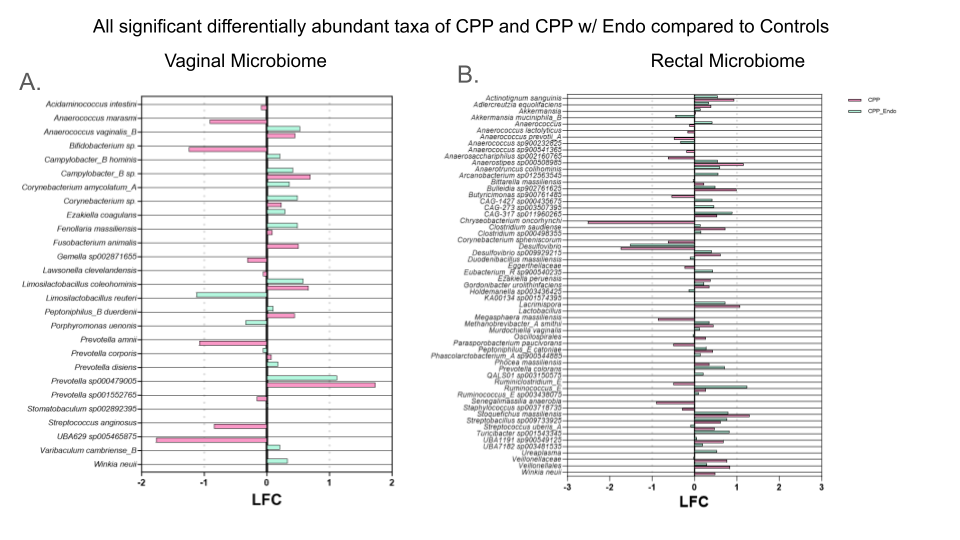


**Figure S4: Differentially abundant taxa of CPP, CPP-Endo compared to surgical controls.** Comparison of differentially abundant taxa of CPP and CPP-Endo vs. Controls with a q value of  < 0.05 with Bonferroni multiple testing correction, indicated by bars in pink for CPP and bars in green for CPP-Endo in both **(A)** vaginal and **(B)** rectal microbiomes. The bacterial enrichment was performed utilizing ANCOM-BC. Pairwise comparisons were from Bonferroni's false discovery adjustment.


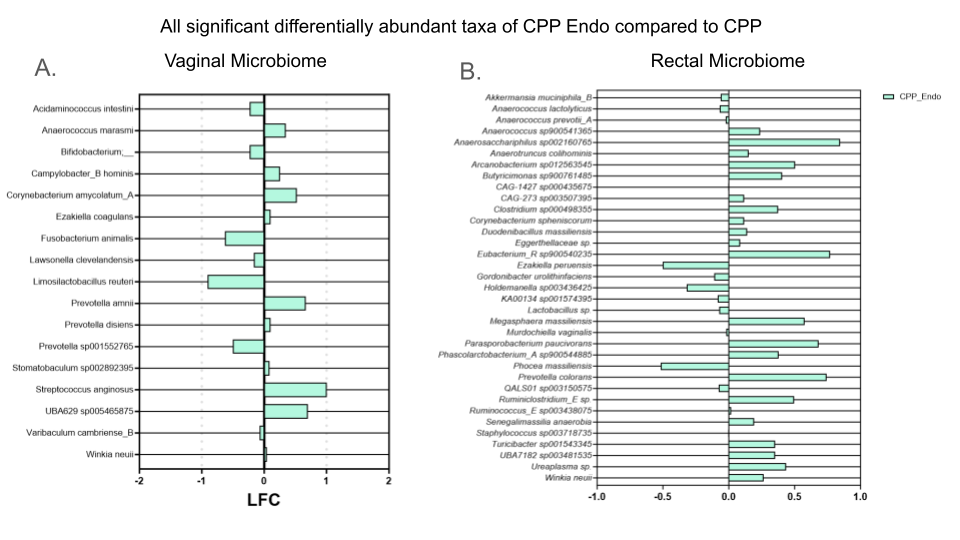


**Figure S5: Differentially abundant taxa of CPP-Endo compared to CPP.** Comparison of differentially abundant taxa of CPP Endo vs. CPP with a q value of  < 0.05 with Bonferroni multiple testing correction and bars in green indicate CPP_Endo for both **(A)** vaginal and **(B)** rectal microbiomes.  The bacterial enrichment was performed utilizing ANCOM-BC.


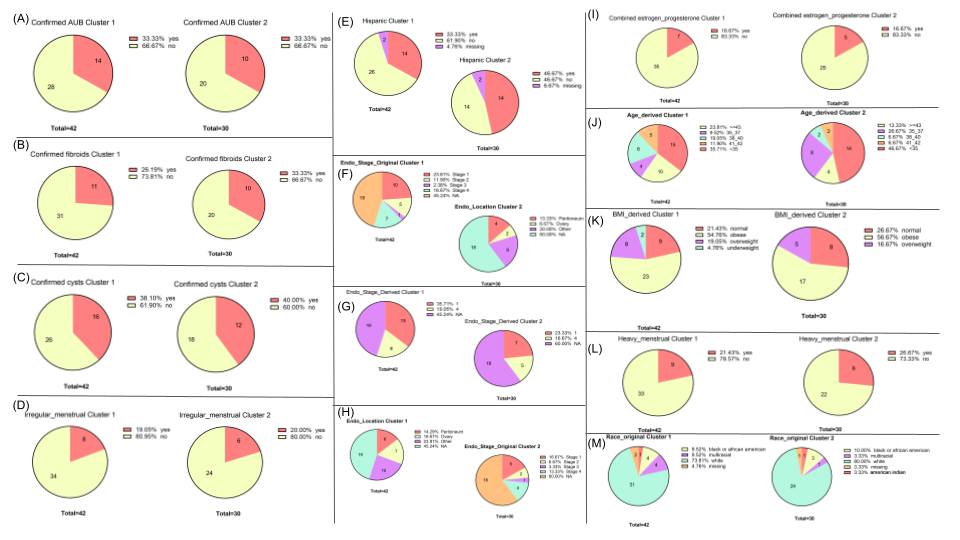


**Figure S6: Cluster metadata analysis from hierarchical heatmap of immune data.** Pie charts show differences observed in distribution between immune clusters 1 and 2 for disease groups **(A)** AUB, **(B)** fibroids, **(C)** ovarian cysts, **(D)** irregular menstrual bleeding, **(E)** Hispanic-ethnicity, **(F)** endometriosis stage ( 1, 2,3,4), **(G)** endometriosis stage derived (½, ¾), **(H)** endometriosis location, **(I)** estrogen and progesterone usage, **(J)** Age, **(K)** BMI, **(L)** heavy menstrual bleeding, and **(M)** Race were not significantly different between the clusters. P-values were calculated using Fisher’s exact test or chi-square test. p-values <0.05 were significant


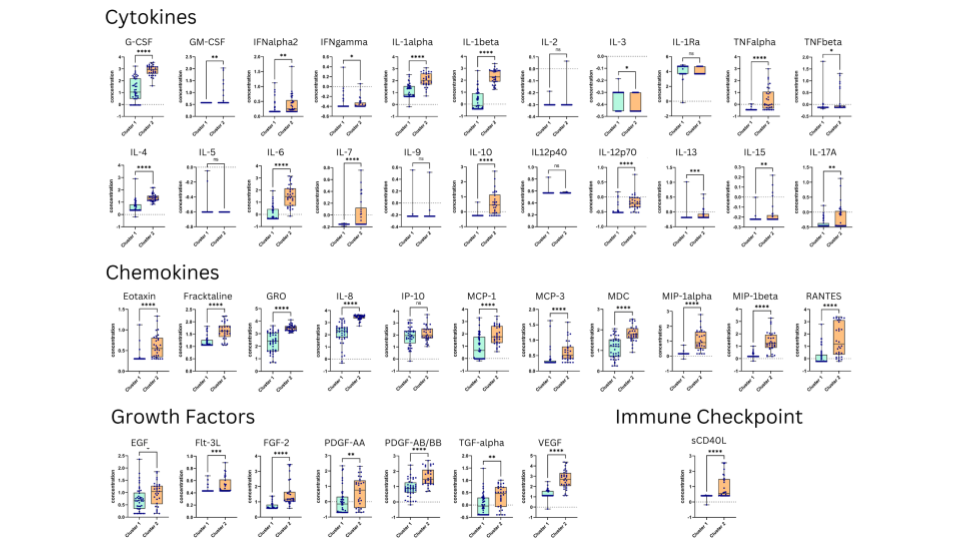


**Figure S7: Immune marker concentration levels amongst distinct clusters identified by hierarchical clustering analysis.** P-values were calculated using a two-sample T-test. P-values <0.05 were significant, where * is denoted as <0.05, ** is denoted as <0.01, *** <0.001, and **** <0.0001 p-value.


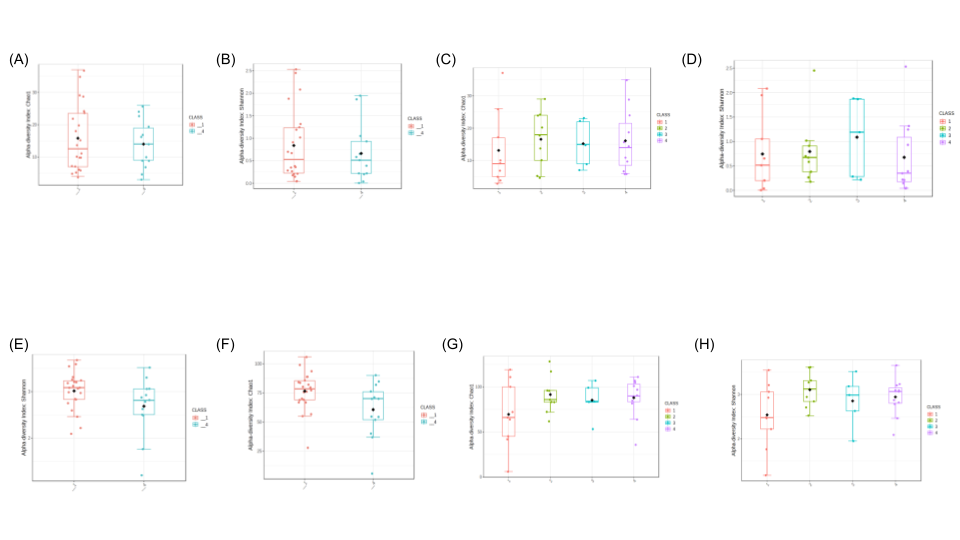


**Figure S8: Microbial alpha-diversity of patients diagnosed with endometriosis by endometriosis stage and location in vaginal and rectal samples. (A)** Boxplots of vaginal alpha-diversity differences between women with endometriosis stage (red) stage 1/2 and (blue) stage 3/4 in vaginal samples at the species level utilizing the Chao1 index (p-value: 0.55193; [T-test] statistic: 0.60137) **(B)** and the Shannon index (p-value: 0.47448; [T-test] statistic: 0.72443); **(C)** and differences between endometriosis location (red) Ovary, (green) peritoneum, (blue) multiple sites, and (purple) other sites in vaginal samples at the species level utilizing the Chao1 index p-value: 0.87657; [ANOVA] F-value: 0.22741, **(D)** and the Shannon index p-value: 0.78944; [ANOVA] F-value: 0.34996. **(E)** Differences between endometriosis stage (red) stage 1/ 2 and (blue) stage 3/4 in rectal samples at the species level utilizing the Shannon index (p-value: 0.065071; [T-test] statistic: 1.9596), **(F)** and the Chao1 index at the species level (p-value: 0.043801; [T-test] statistic: 2.1576); and **(G)** differences between endometriosis location (red) ovary, (green) peritoneum, (blue) multiple sites, and (purple) other sites in rectal samples at the species level utilizing the Shannon index (p-value: 0.17752; [ANOVA] F-value: 1.7525), **(H)** and the Chao1 index at the species level (p-value: 0.3063; [ANOVA] F-value: 1.2584).


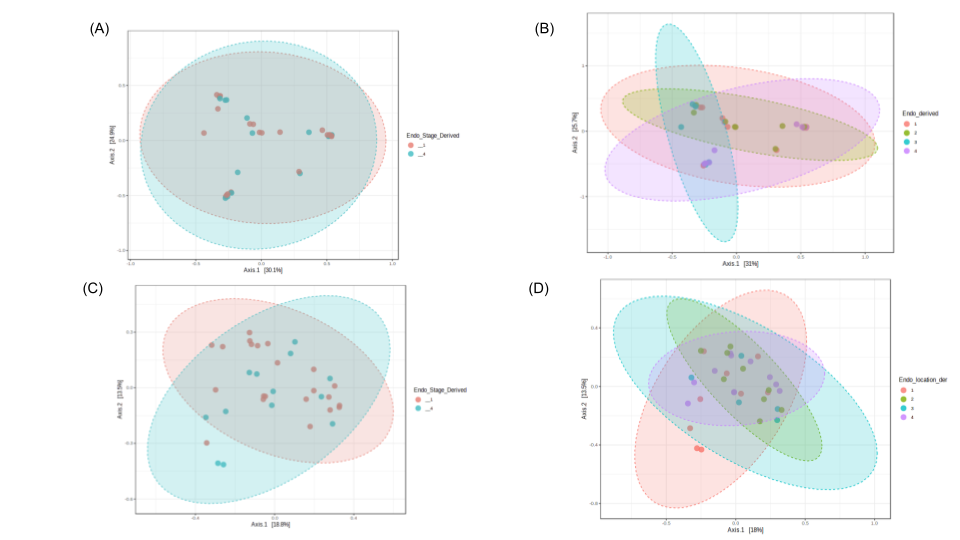


**Figure S9: Microbial beta-diversity of patients diagnosed with endometriosis by endometriosis stage and location in vaginal and rectal samples. (A)** A beta-diversity measure demonstrates dissimilarity of taxa in women with endometriosis stage (red) stage 1 and stage 2 and (blue) stage 3 and stage 4 in vaginal samples at the species level utilizing the Bray-Curtis index ([PERMANOVA] F-value: 0.39478; R-squared: 0.011822; p-value: 0.87); **(B)** differences between endometriosis location (red) Ovary, (green) peritoneum, (blue) multiple sites, and (purple) other sites in vaginal samples at the species level utilizing the Bray-Curtis index ([PERMANOVA] F-value: 1.5127; R-squared: 0.1314; p-value: 0.127). **(C)** Differences in endometriosis stage (red) stage 1 and stage 2 and (blue) stage 3 and stage 4 in rectal samples at the species level utilizing the Bray-Curtis index ([PERMANOVA] F-value: 0.92162; R-squared: 0.027169; p-value: 0.524); **(D)** differences between (red) Ovary, (green) peritoneum, (blue) multiple sites, and (purple) other sites in rectal samples at the species level utilizing the Bray-Curtis index ([PERMANOVA] F-value: 1.0151; R-squared: 0.092154; p-value: 0.412). Each point represents a sample.


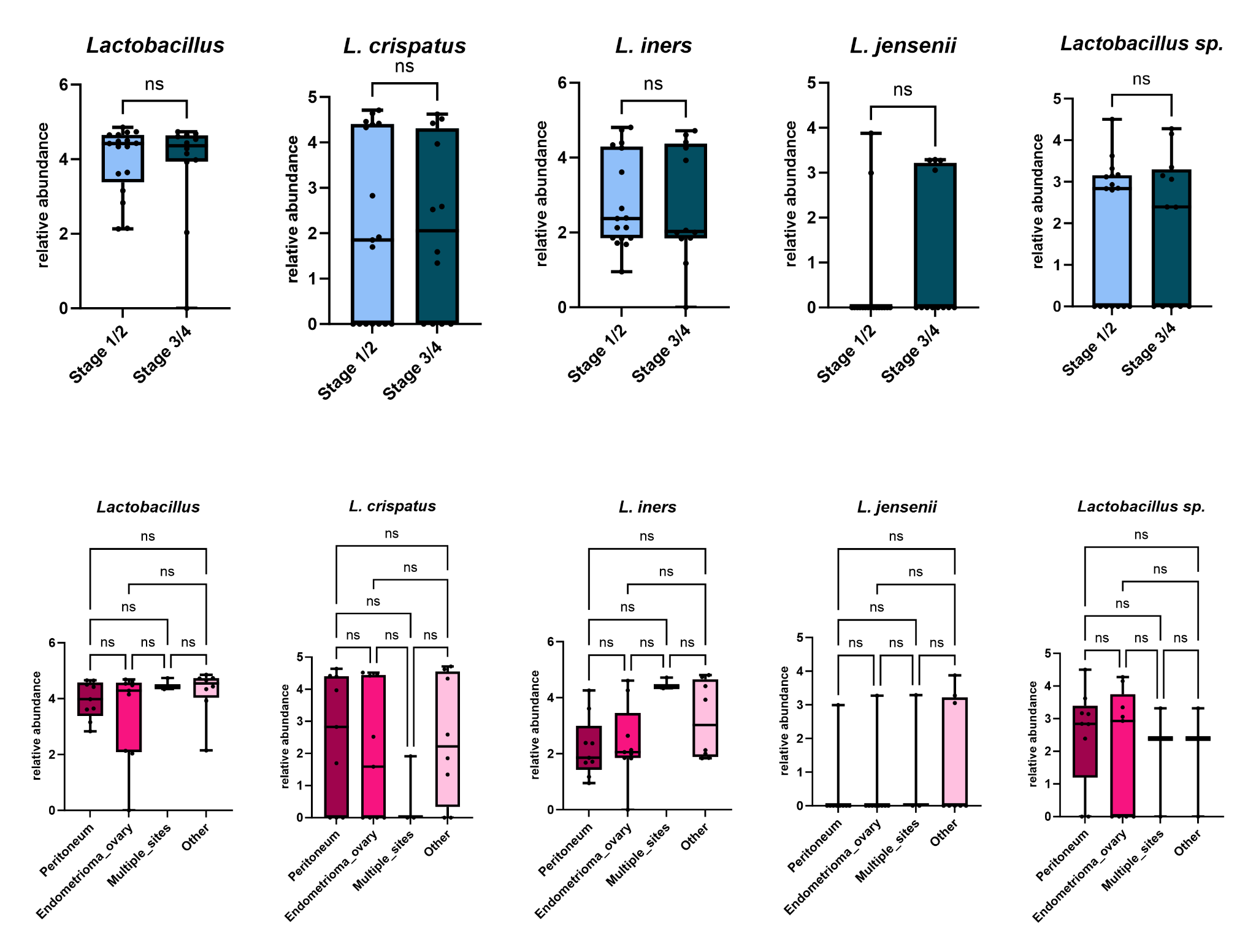


**Figure S10: Vaginal *Lactobacillus* abundances across endometriosis stage and location.** Abundance plots of Lactobacillus genus and species amongst endometriosis characteristics. **(A)** Box plots of Lactobacillus genus and species log10 transformed amongst patients diagnosed with stage 1/2 endometriosis (light blue) and stage 3/4 endometriosis (dark blue). **(B)** Box plots of Lactobacillus genus and species log10 transformed amongst sites of endometriosis with peritoneum (dark red), endometrioma/ovary (pink), multiple locations (red), and other locations such as bowel or bladder (light pink). Additional abundance difference testing is performed by the Mann-Whitney test where “ns” is not significant, * is <0.05,  ** is <0.01, *** <0.001, and **** <0.0001 p-value.


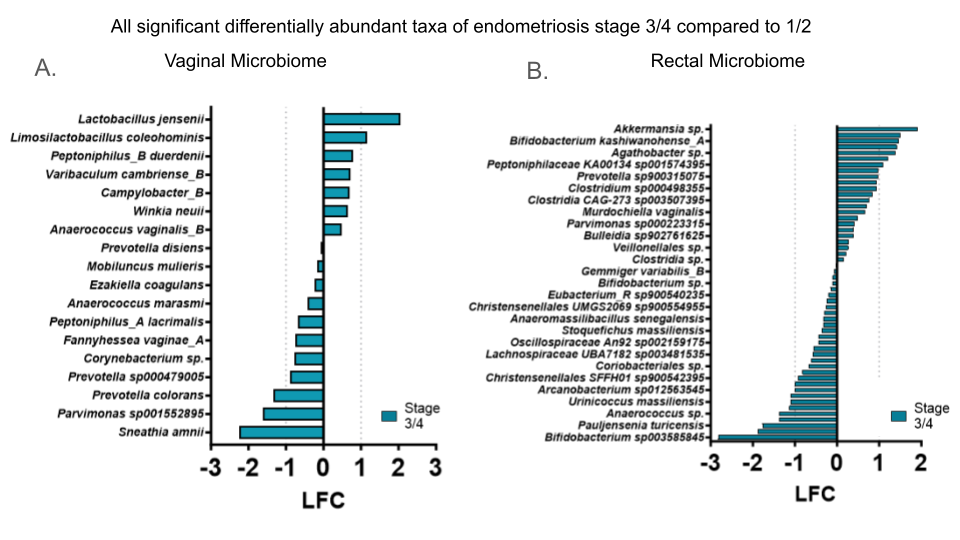


**Figure S11: Differentially abundant taxa of endometriosis stage 3/4 compared to endometriosis stage 1/2.** Comparison of differentially abundant taxa of endometriosis stage 3/4 versus endometriosis stage 1/2 with q value of < 0.05 with Bonferroni multiple testing correction. Bars in blue indicate endometriosis stage 3/4 for (**A**) vaginal and (**B**) rectal microbiomes The bacterial enrichment was performed utilizing ANCOM-BC.


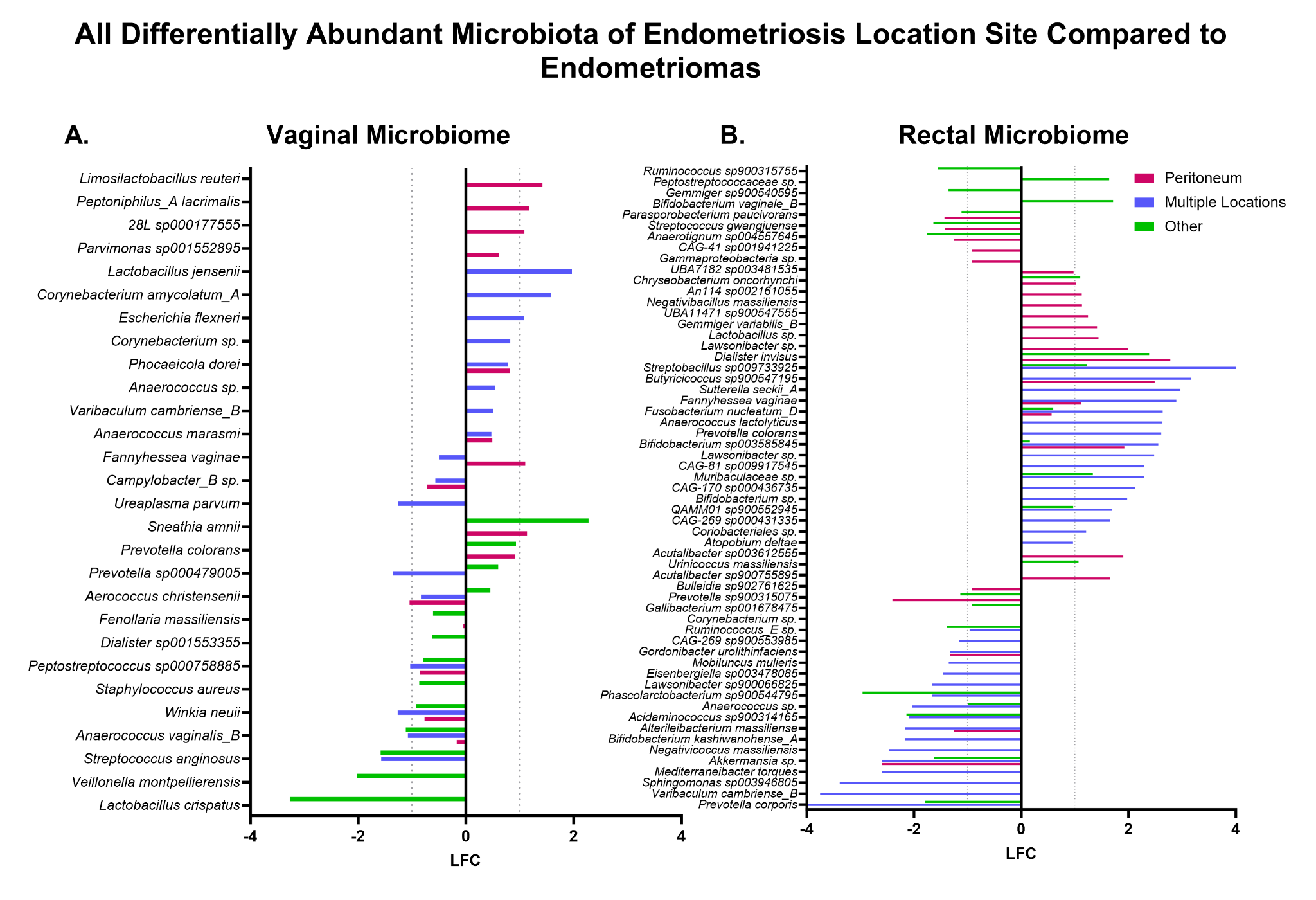


**Figure S12: Differentially abundant taxa of endometriosis location sites.** Comparison of differentially abundant taxa of the peritoneum, other locations, and multiple locations compared to endometrioma/ovary with a q-value of < 0.05 by Bonferroni multiple testing correction. Bars indicate peritoneum, other, and multiple site log-fold change differences compared to the ovary. Analysis was performed in both (**A**) vaginal and (**B**) rectal microbiomes. The bacterial enrichment was performed utilizing ANCOM-BC.


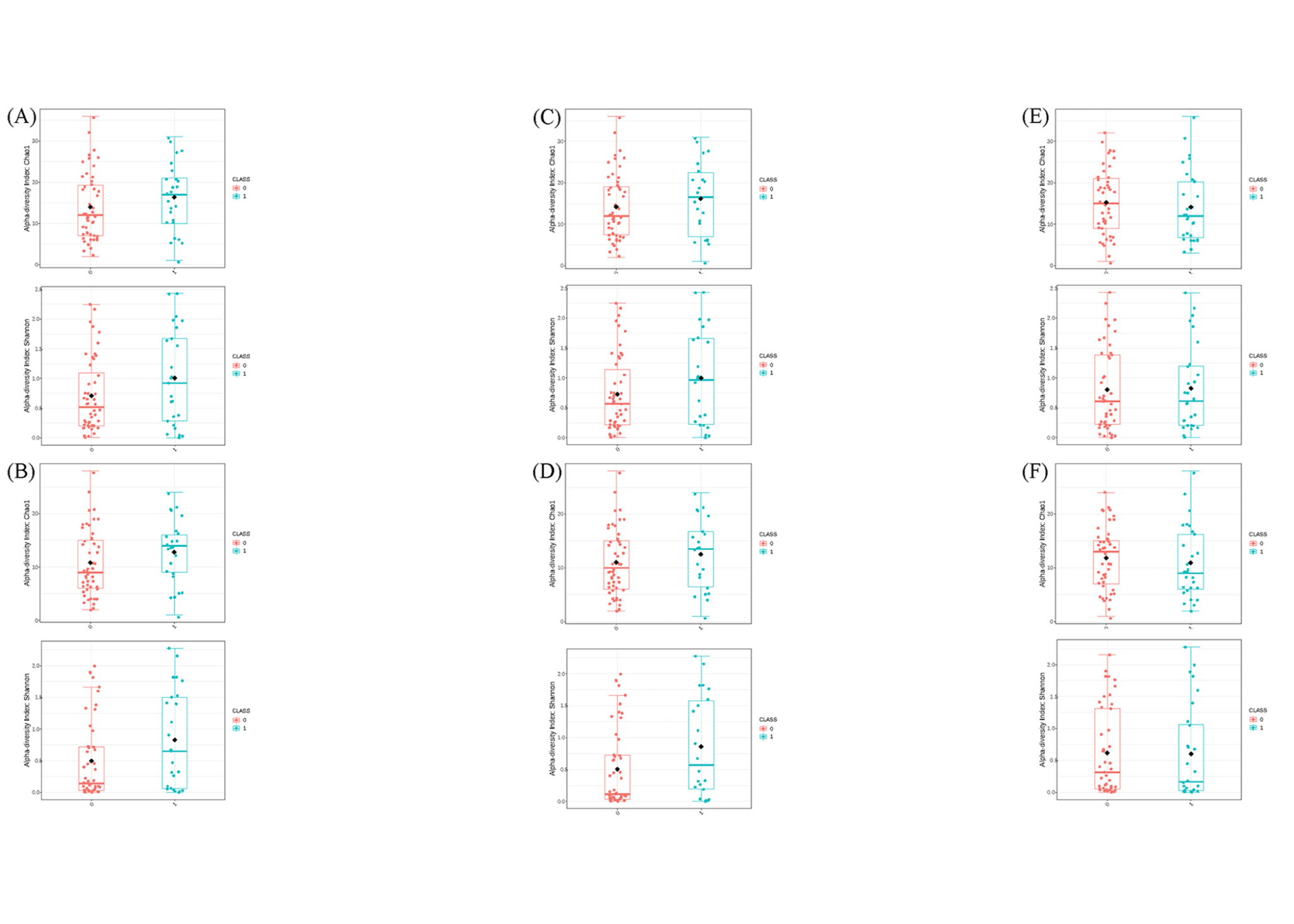


**Figure S13: Microbial alpha-diversity of patients diagnosed with abnormal uterine bleeding, fibroids, and ovarian cysts in vaginal samples.** Boxplots of vaginal alpha-diversity differences between women with (blue) and without (red) diagnosed co-occurring gynecologic conditions. **(A)** abnormal uterine bleeding (AUB) at the species level utilizing the Chao1 index (top) (p-value: 0.25685; [T-test] statistic: -1.1475) and the Shannon index (bottom) (p-value: 0.11579; [T-test] statistic: -1.6085); **(B)** and at the genus level utilizing the Chao1 index (top) (p-value: 0.19945; [T-test] statistic: -1.3004) and the Shannon index (bottom) (p-value: 0.07303; [T-test] statistic: -1.84). **(C)** fibroids at the species level utilizing the Chao1 index(top) (p-value: 0.0.3838; [T-test] statistic: -0.8817) and the Shannon index (bottom) (p-value: 0.17745; [T-test] statistic: -1.3789); **(D)** and at the genus level utilizing the Chao1 index (top) (p-value: 0.36728; [T-test] statistic: -0.91265) and the Shannon index (bottom) (p-value: 0.070047; [T-test] statistic: -1.8725). **(E)** ovarian cysts (cysts) at the species level utilizing the Chao1 index (top) (p-value: 0.60176; [T-test] statistic: 0.52499) and the Shannon index (bottom) (p-value: 0.89616; [T-test] statistic: -0.13111); **(F)** and at the genus level utilizing the Chao1 index (top) (p-value: 0.56176; [T-test] statistic: 0.58403) and the Shannon index (bottom) (p-value: 0.91402; [T-test] statistic: 0.10848).


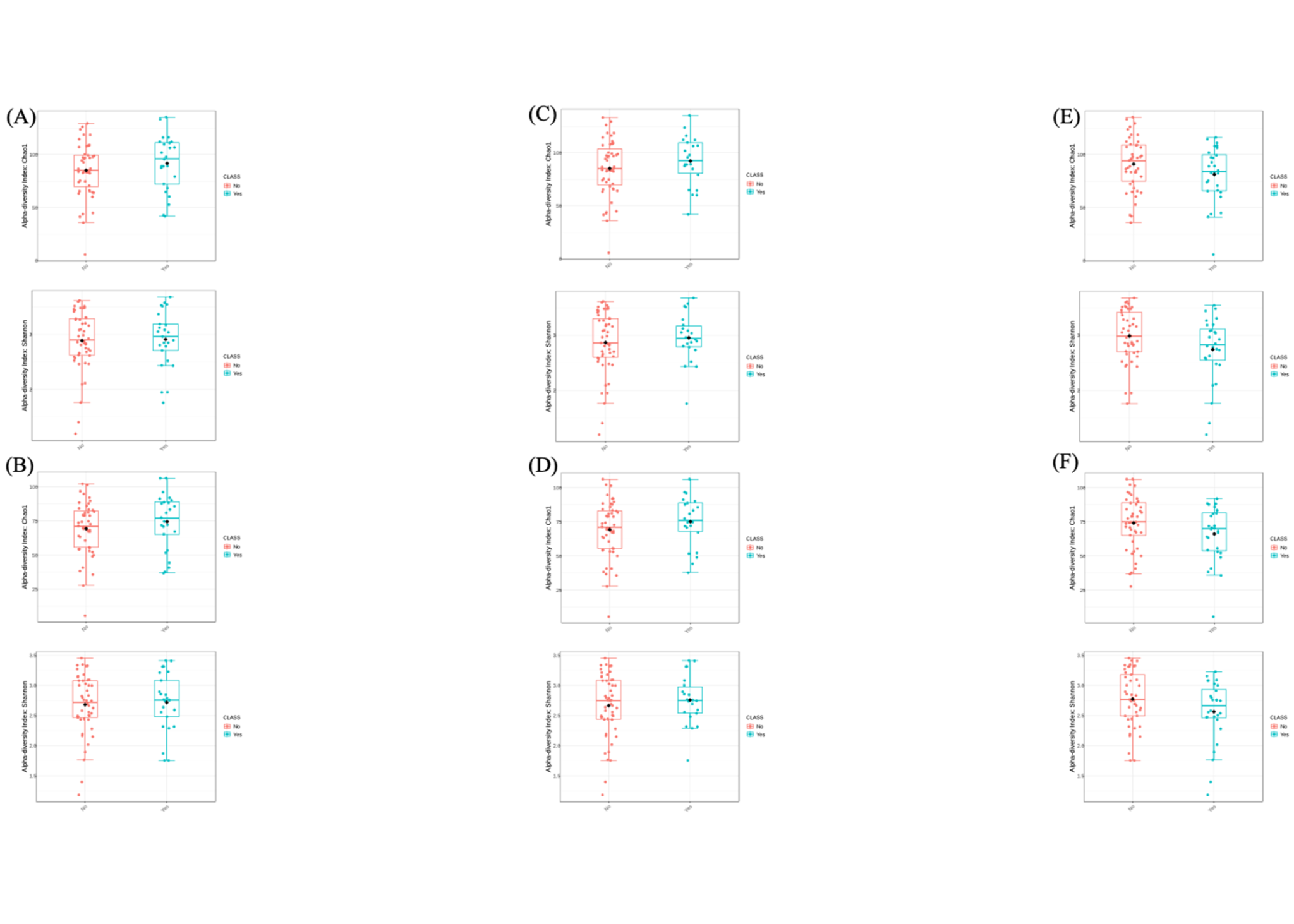


**Figure S14: Microbial alpha-diversity of patients diagnosed with abnormal uterine bleeding, fibroids, and ovarian cysts in rectal samples.** Boxplots of rectal alpha-diversity differences between women with (blue) and without (red) diagnosed co-occurring gynecologic conditions. **(A)** abnormal uterine bleeding (AUB) at the species level utilizing the Chao1 index (top) (p-value: 0.3029; [T-test] statistic: -1.0415) and the Shannon index (bottom) (p-value: 0.85471; [T-test] statistic: -0.18405); **(B)** and at the genus level utilizing the Chao1 index (top) (p-value: 0.30863; [T-test] statistic: -1.0293) and the Shannon index (bottom) (p-value: 0.75711; [T-test] statistic: -0.31096). **(C)** fibroids at the species level utilizing the Chao1 index (top) (p-value: 0.26619; [T-test] statistic: -1.126) and the Shannon index (bottom) (p-value: 0.47547; [T-test] statistic: -0.71894); **(D)** and at the genus level utilizing the Chao1 index (top) (p-value: 0.24371; [T-test] statistic: -1.1816) and the Shannon index (bottom) (p-value: 0.41803; [T-test] statistic: -0.81652). **(E)** ovarian cysts (cysts) at the species level utilizing the Chao1 index (top) (p-value: 0.1136; [T-test] statistic: 1.6076) and the Shannon index (bottom) (p-value: 0.67696; [T-test] statistic: 1.8691); **(F)** and at the genus level utilizing the Chao1 index (top) (p-value: 0.096925; [T-test] statistic: 1.6881) and the Shannon index (bottom) (p-value: 0.084252; [T-test] statistic: 1.7598).


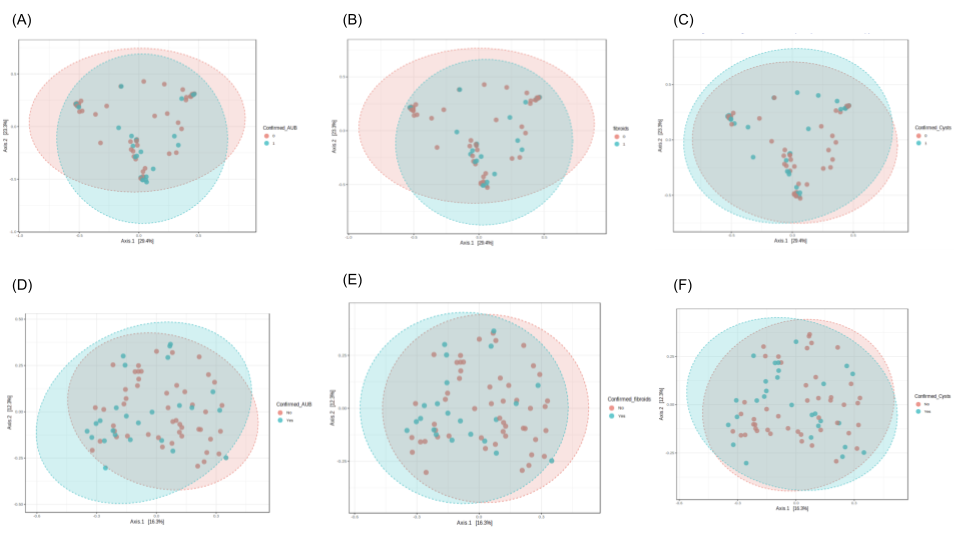


**Figure S15: Microbial beta-diversity of patients diagnosed with abnormal uterine bleeding, fibroids, and ovarian cysts in vaginal and rectal samples.** A beta-diversity measure demonstrates the dissimilarity of taxa at the species level in women with (blue) and without (red) diagnosed co-occurring gynecologic conditions utilizing the Bray-Curtis index. Vaginal samples **(A)** abnormal uterine bleeding (AUB) ([PERMANOVA] F-value: 1.1779; R-squared: 0.016319; p-value: 0.317); **(B)** fibroids ([PERMANOVA] F-value: 1.579; R-squared: 0.021756; p-value: 0.173); **(C)** ovarian cysts (cysts) ([PERMANOVA] F-value: 0.59033; R-squared: 0.0082459; p-value: 0.683). Rectal samples **(D)** abnormal uterine bleeding (AUB) ([PERMANOVA] F-value: 1.5145; R-squared: 0.020885; p-value: 0.1); **(E)** fibroids ([PERMANOVA] F-value: 1.0832; R-squared: 0.015027; p-value: 0.338); **(F)** ovarian cysts (cysts) ([PERMANOVA] F-value: 1.2768; R-squared: 0.017666; p-value: 0.19). Each point represents a sample.


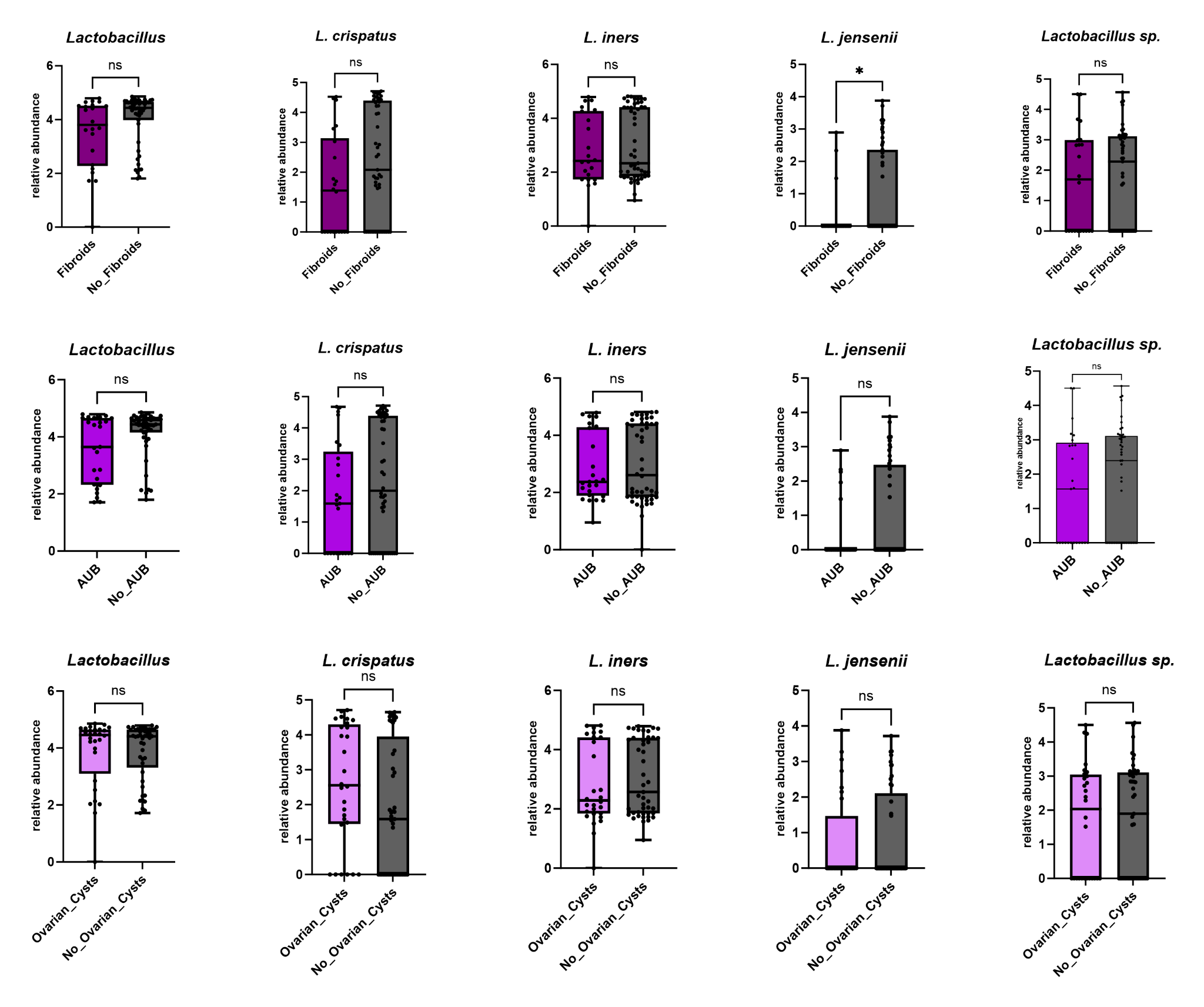


**Figure S16: Vaginal *Lactobacillus* abundances across co-occurring gynecologic conditions.** Abundance plots of *Lactobacillus* genus and species amongst other benign gynecologic conditions within the CPP cohort. **(A)** Box plots of *Lactobacillus* genus and species log10 transformed amongst patients diagnosed with fibroids (dark purple) and no fibroids(gray). **(B)** Box plots of *Lactobacillus* genus and species log10 transformed amongst patients diagnosed with abnormal uterine bleeding/AUB (purple) and no AUB (gray). **(C)** Box plots of *Lactobacillus* genus and species log10 transformed amongst patients diagnosed with ovarian cysts (light purple) and no ovarian cysts (gray). Additional abundance difference testing is performed by the Mann-Whitney test where “**ns**” is not significant, * is <0.05, ** is <0.01, *** <0.001, and **** <0.0001 p-value.


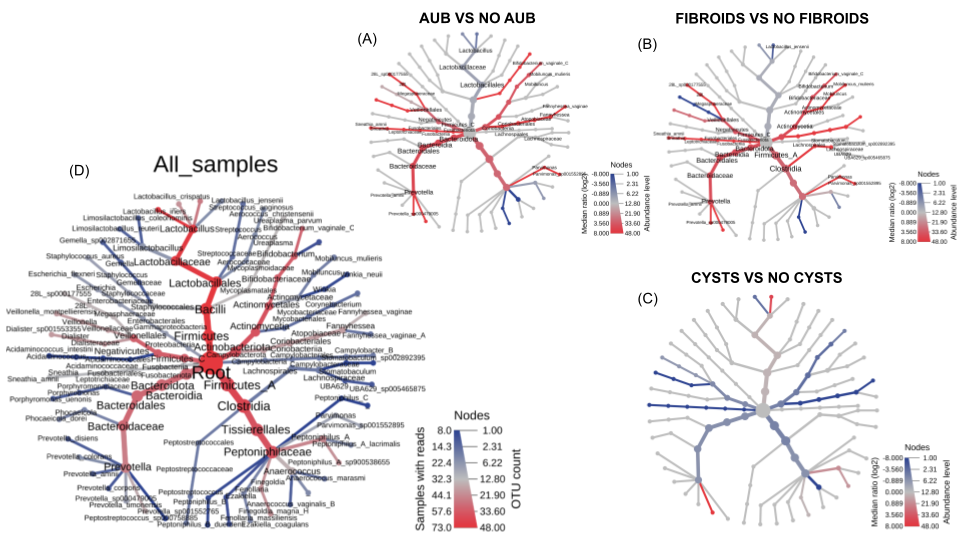


**Figure S17: HeatTree identifies differentially abundant taxa amongst co-occurring conditions: Abnormal uterine bleeding, fibroids, and ovarian cysts in vaginal samples.**

Heat tree analysis depicts the hierarchical structure of taxonomic classification with median abundance and statistical taxonomic differences in microbial composition in women with and without diagnosed co-occurring gynecologic conditions. Wilcoxon Rank Sum test is used to determine statistically significant vaginal taxa (*p*= 0.05), **(A)** in women with abnormal uterine bleeding (AUB) versus women without AUB, **(B)** in women with fibroids versus women without fibroids, **(C)** in women with ovarian cysts (cysts) versus women without cysts. **(D)** depicts the hierarchical structure of the taxonomic classification of taxa in all samples for reference.


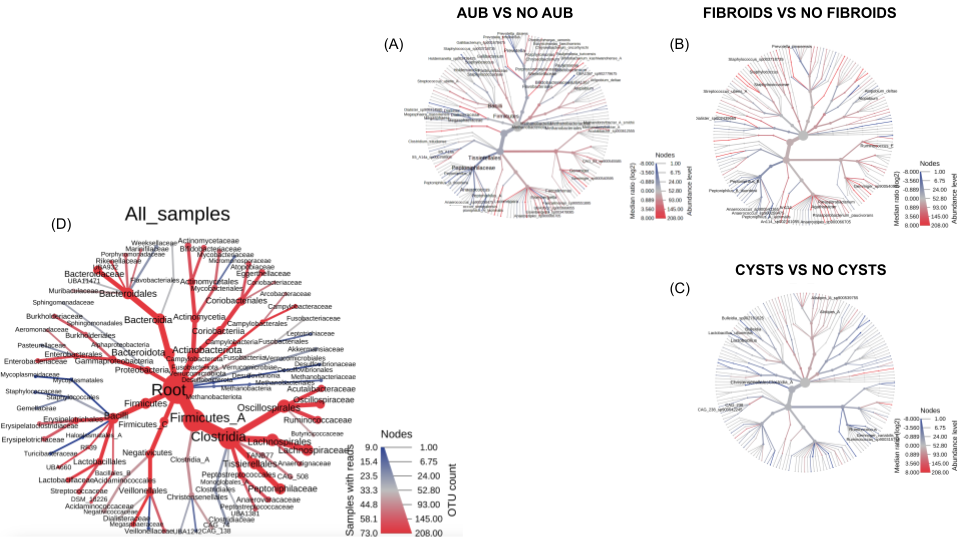


**Figure S18: HeatTree identifies differentially abundant taxa amongst co-occurring conditions: Abnormal uterine bleeding, fibroids, and ovarian cysts in rectal samples.**

Heat tree analysis depicts the hierarchical structure of taxonomic classification with median abundance and statistical taxonomic differences in microbial composition in women with and without diagnosed co-occurring gynecologic conditions. Wilcoxon Rank Sum test is used to determine statistically significant rectal taxa (*p*= 0.05), **(A)** in women with abnormal uterine bleeding (AUB) versus women without AUB, **(B)** in women with fibroids versus women without fibroids, **(C)** in women with ovarian cysts (cysts) versus women without cysts. **(D)** depicts the hierarchical structure of the taxonomic classification of taxa in all samples for reference.


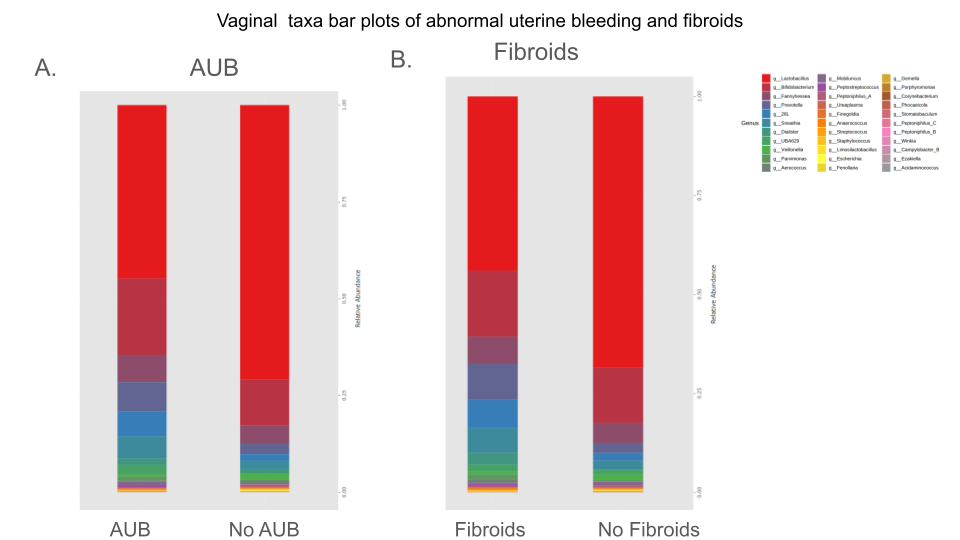


**Figure S19: Vaginal taxa profiles of co-occurring condition groups in the CPP cohort.** The microbiome profile is significantly different between patients diagnosed with co-occurring conditions of AUB and fibroids compared to patients with no AUB and no fibroids. No taxa were differentially abundant in ovarian cysts and no ovarian cyst groups; therefore, global profiles were not visualized. Grouped taxa barplot of genera in vaginal microbiomes across co-occurring condition groups: **(A)** abnormal uterine bleeding (AUB) and no abnormal uterine bleeding (AUB), **(B)** Fibroids and no fibroids.


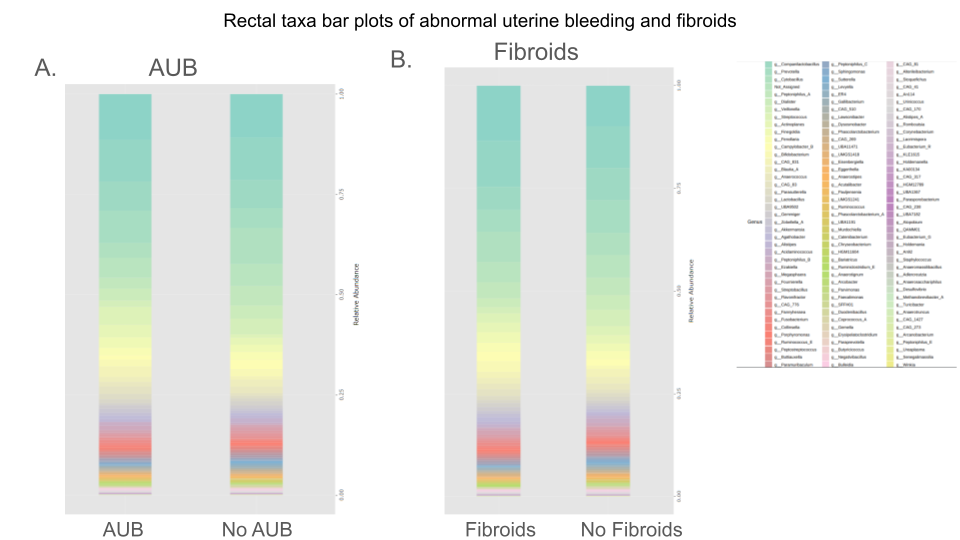


**Figure S20: Gut taxa profiles of co-occurring condition groups in the CPP cohort.** The microbiome profile is significantly different between patients diagnosed with co-occurring conditions of AUB and fibroids compared to patients with no AUB and no fibroids. No taxa were differentially abundant in ovarian cysts, and no ovarian cyst groups; therefore, global profiles were not visualized. Grouped taxa barplot of genera in gut microbiomes across co-occurring condition groups: **(A)** abnormal uterine bleeding (AUB) and no abnormal uterine bleeding (AUB), **(B)** Fibroids and no fibroids.


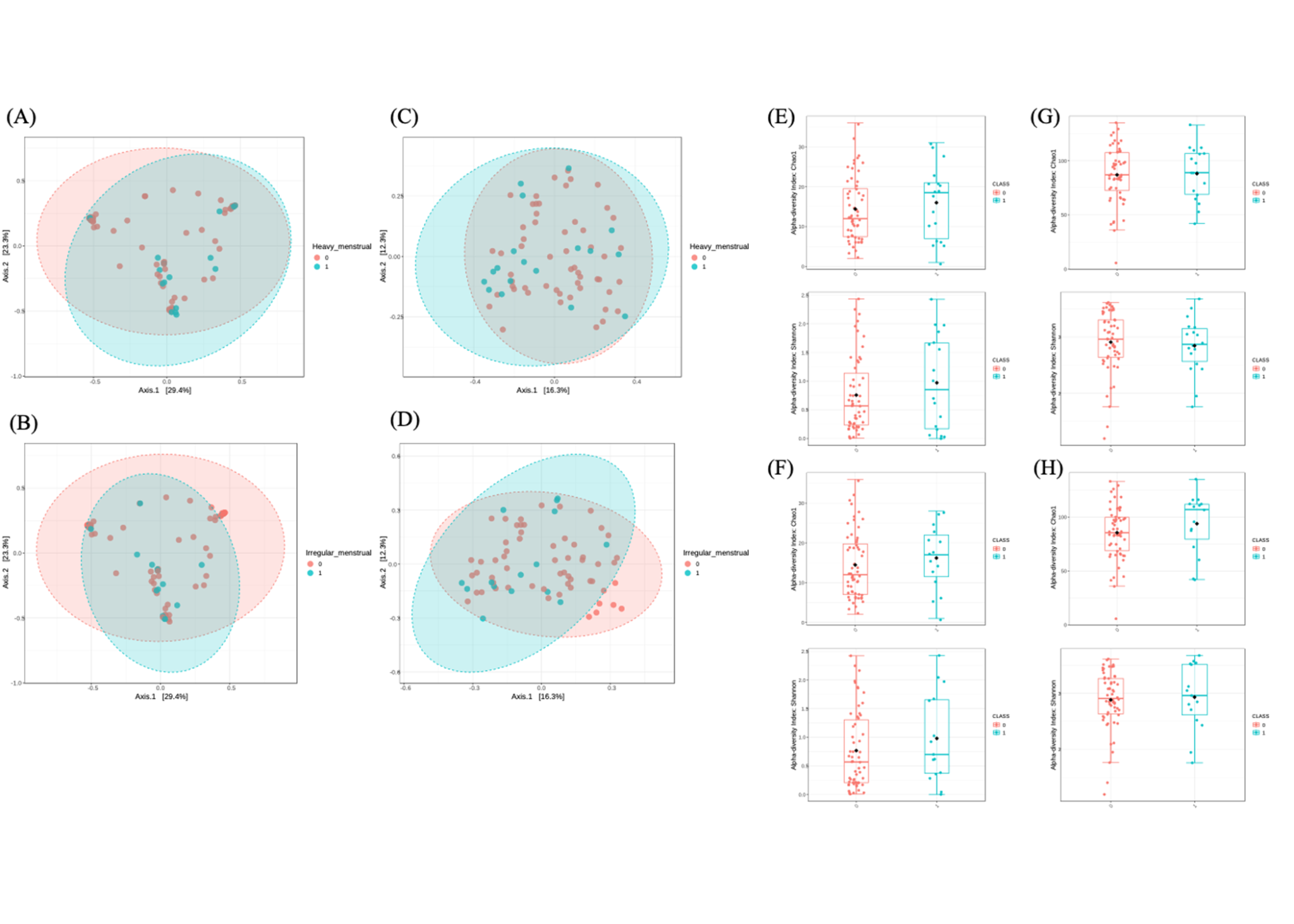


**Figure S21: Microbial diversity of patients diagnosed with heavy menstrual bleeding and irregular menstrual bleeding in vaginal and rectal samples.** A beta-diversity measure demonstrates dissimilarity of taxa at the species level in women with (blue) and without (red) diagnosed abnormal uterine bleeding (heavy bleeding and irregular bleeding) utilizing the Bray-Curtis index. Vaginal samples **(A)** heavy bleeding ([PERMANOVA] F-value: 1.1479; R-squared: 0.015911; p-value: 0.302); **(B)** irregular bleeding ([PERMANOVA] F-value: 0.57182; R-squared: 0.0079894; p-value: 0.703). Rectal samples **(C)** heavy bleeding ([PERMANOVA] F-value: 0.90512; R-squared: 0.012588; p-value: 0.541); **(D)** irregular bleeding ([PERMANOVA] F-value: 1.2196; R-squared: 0.016887; p-value: 0.237). Each point represents a sample. Boxplots of alpha-diversity differences between women with (blue) and without (red) diagnosed abnormal uterine bleeding (heavy bleeding and irregular bleeding). Vaginal samples **(E)** heavy bleeding at the species level utilizing the Chao1 index (top) (p-value: 0.51957; [T-test] statistic: -0.65282) and the Shannon index (bottom) (p-value: 0.32538; [T-test] statistic: -1.0037); **(F)** irregular bleeding at the species level utilizing the Chao1 index (top) (p-value: 0.46975; [T-test] statistic: -0.73544) and the Shannon index (bottom) (p-value: 0.34712; [T-test] statistic: -0.96307). Rectal samples **(G)** heavy bleeding at the species level utilizing the Chao1 index (top) (p-value: 0.86768; [T-test] statistic: -0.16799) and the Shannon index (bottom) (p-value: 0.645; [T-test] statistic: 0.46522); **(H)** irregular bleeding at the species level utilizing the Chao1 index (top) (p-value: 0.3078; [T-test] statistic: -1.0465) and the Shannon index (bottom) (p-value: 0.77566; [T-test] statistic: -0.28889).


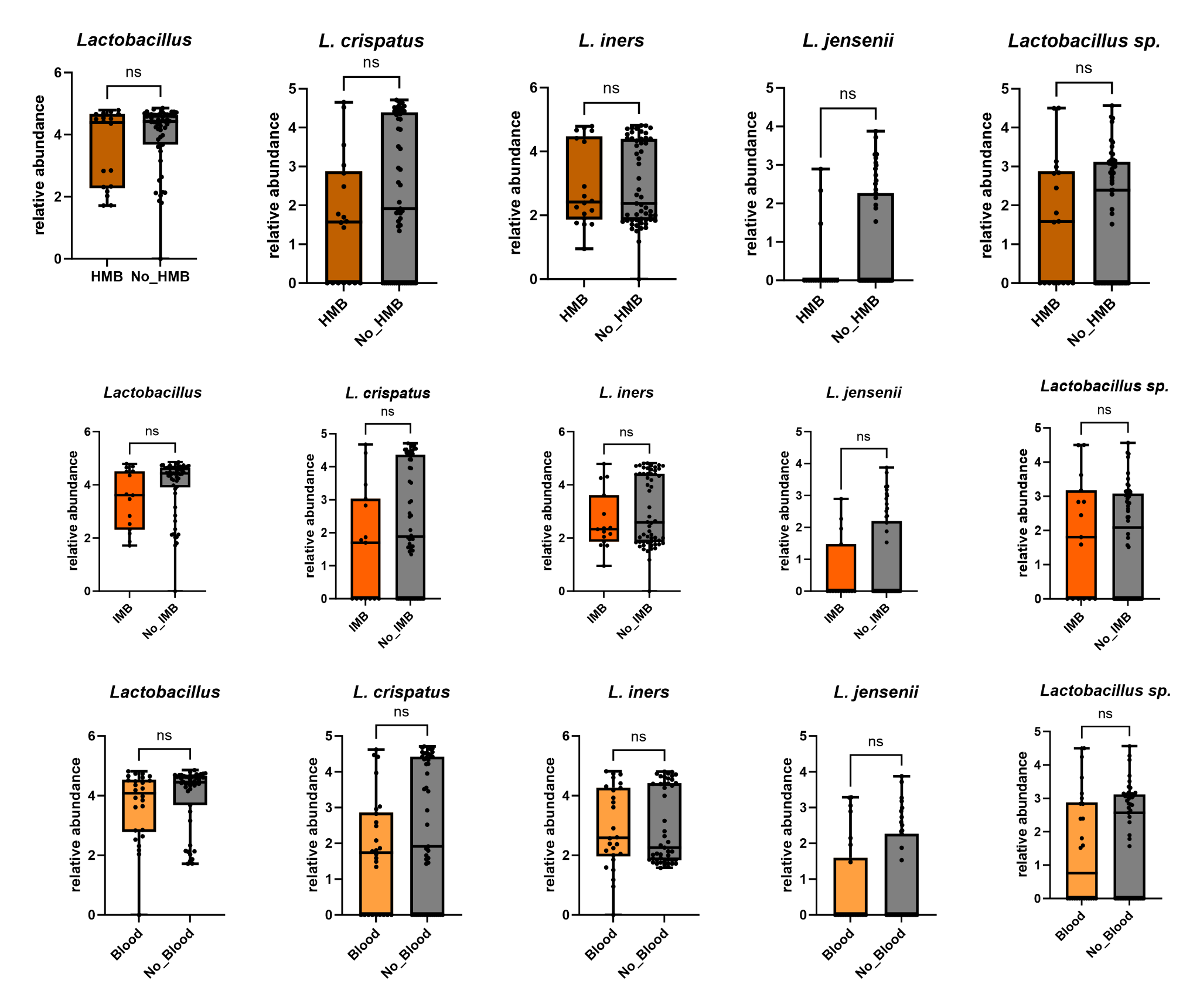


**Figure S22: Vaginal *Lactobacillus* abundances across menstrual bleeding symptoms.** Abundance plots of *Lactobacillus* genus and species amongst menstrual bleeding symptoms and blood in cervical, vaginal samples within CPP cohort. **(A)** Box plots of *Lactobacillus* genus and species log10 transformed amongst patients diagnosed with heavy menstrual bleeding (HMB) (dark orange) and no heavy menstrual bleeding (no HMB) (gray). **(B)** Box plots of *Lactobacillus* genus and species log10 transformed amongst patients with irregular menstrual bleeding (IMB) (orange) and no irregular menstrual bleeding (No IMB) (gray). **(C)** Box plots of *Lactobacillus* genus and species log10 transformed amongst patient samples with blood (light orange) and no blood (gray). Additional abundance difference testing is performed by the Mann-Whitney test where “**ns**” is not significant, * is <0.05, ** is <0.01, *** <0.001, and **** <0.0001 p-value.


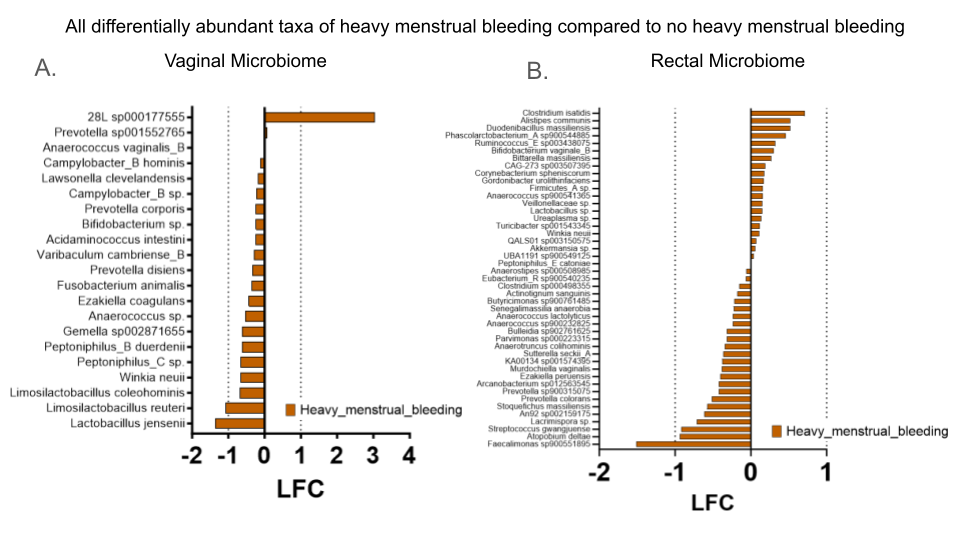


**Figure S23: Differentially abundant taxa of heavy menstrual bleeding.** Comparison of differentially abundant taxa of the symptom of heavy menstrual bleeding and no heavy menstrual bleeding with a q-value of < 0.05 by Bonferroni multiple testing correction. Bars indicate peritoneum, other, and multiple site log-fold change differences compared to the ovary. Analysis was performed in both (**A**) vaginal and (**B**) rectal microbiomes. The bacterial enrichment was performed utilizing ANCOM-BC.


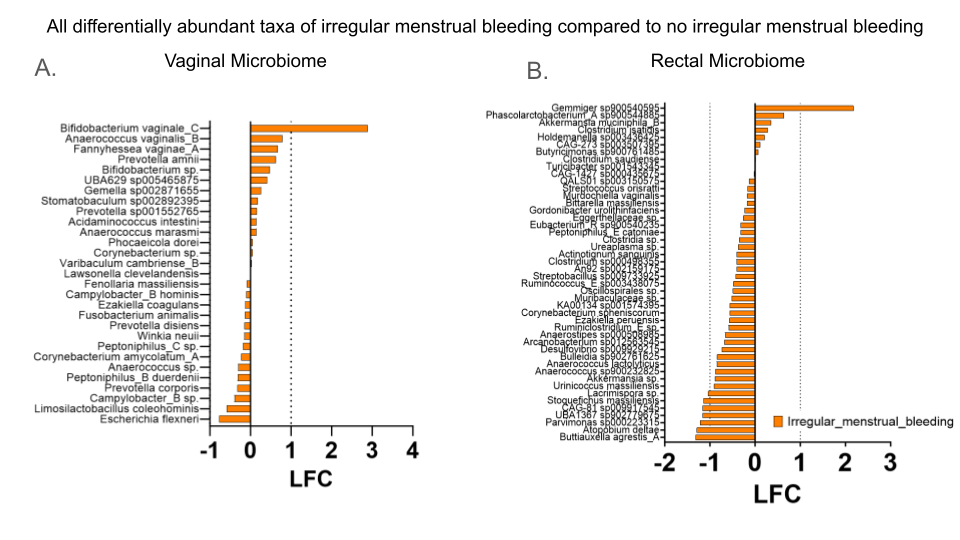


**Figure S24: Differentially abundant taxa of irregular menstrual bleeding.** Comparison of differentially abundant taxa of the symptom of irregular menstrual bleeding and no irregular menstrual bleeding with a q-value of < 0.05 by Bonferroni multiple testing correction. Bars indicate peritoneum, other, and multiple site log-fold change differences compared to the ovary. Analysis was performed in both (**A**) vaginal and (**B**) rectal microbiomes. The bacterial enrichment was performed utilizing ANCOM-BC.


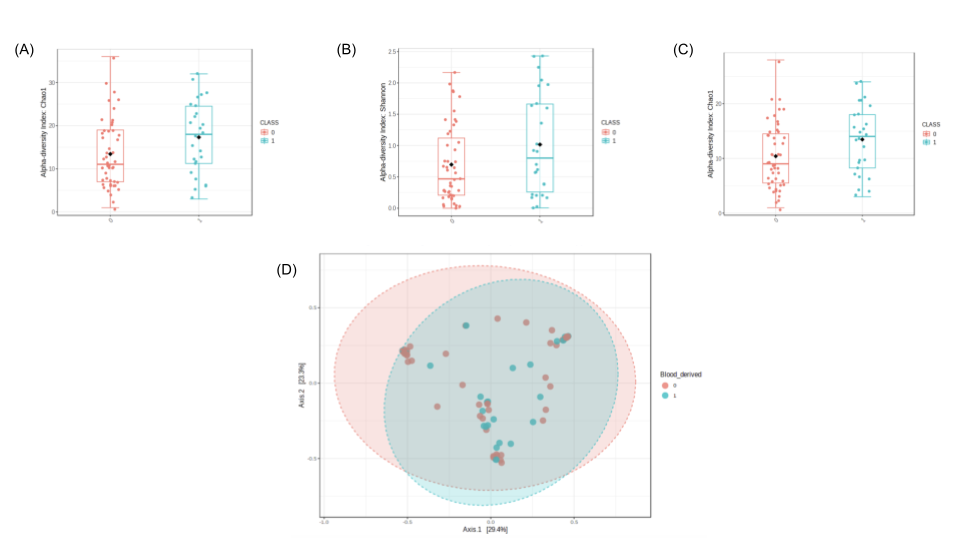


**Figure S25: Microbial diversity of blood present in vaginal samples.**

Boxplots of vaginal alpha-diversity differences between women with (blue) and without (red) blood present in vaginal samples **(A)** at the species level utilizing the Chao1 index (p-value: 0.061901; [T-test] statistic: -1.9106) **(B)** and the Shannon index (p-value: 0.086917; [T-test] statistic: -1.754); **(C)** and at the genus level utilizing the Chao1 index (p-value:0.049834; [T-test] statistic: -2.0103). A beta-diversity measure demonstrates the dissimilarity of vaginal taxa at the species level in women with (blue) and without (red) blood present in vaginal samples **(D)** at the species level utilizing the Bray-Curtis index ([PERMANOVA] F-value 1.0383; R-squared: 0.014414; p-value: 0.375). Each point represents a sample.


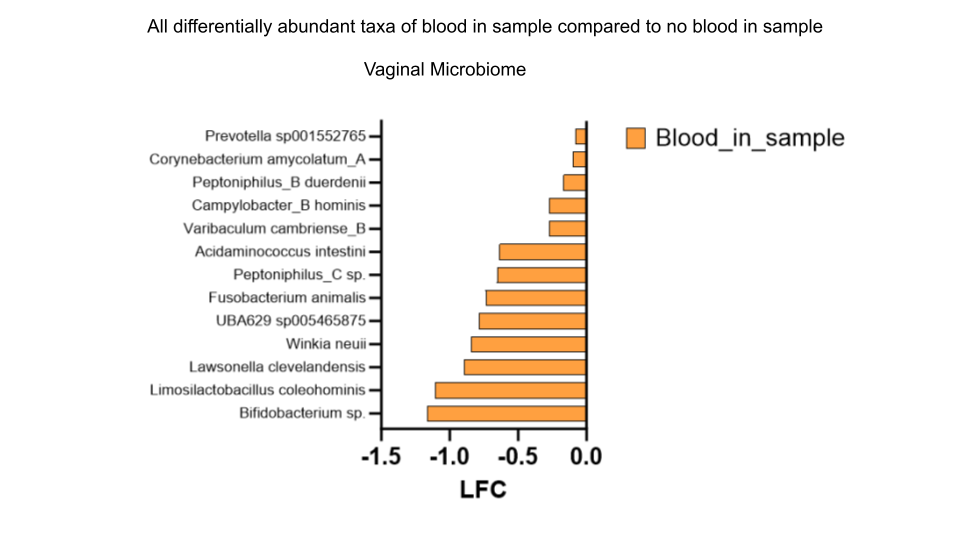


**Figure S26: Differentially abundant taxa of blood observed in the vaginal sample.** Comparison of differentially abundant taxa of the peritoneum, other locations, and multiple locations compared to endometrioma/ovary with a q-value of < 0.05 by Bonferroni multiple testing correction. Bars indicate peritoneum, other, and multiple site log-fold change differences compared to the ovary. Analysis was performed in vaginal microbiomes as samples were collected from the vagina and would be potentially affected by the presence of blood. The bacterial enrichment was performed utilizing ANCOM-BC.


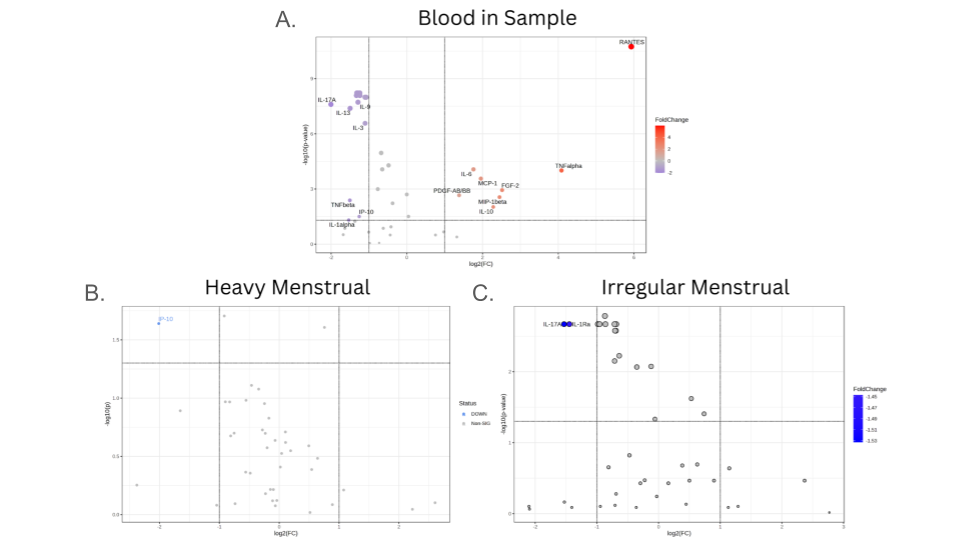


**Figure S27: Immune Protein concentrations comparisons of heavy and irregular menstrual bleeding and blood within the sample.** A volcano plot was used to visualize differences in the immune protein levels among patients based on the menstrual bleeding and blood groups. Statistical significance was determined using a two-sample t-test with the false discovery rate (FDR) correction proteins with q < 0.05 were considered significant. Comparison of protein biomarkers in groups of **(A)** blood in the sample compared to no blood in the sample, **(B)** heavy menstrual compared to no heavy menstrual, and **(C)** irregular menstrual compared to no irregular menstrual.


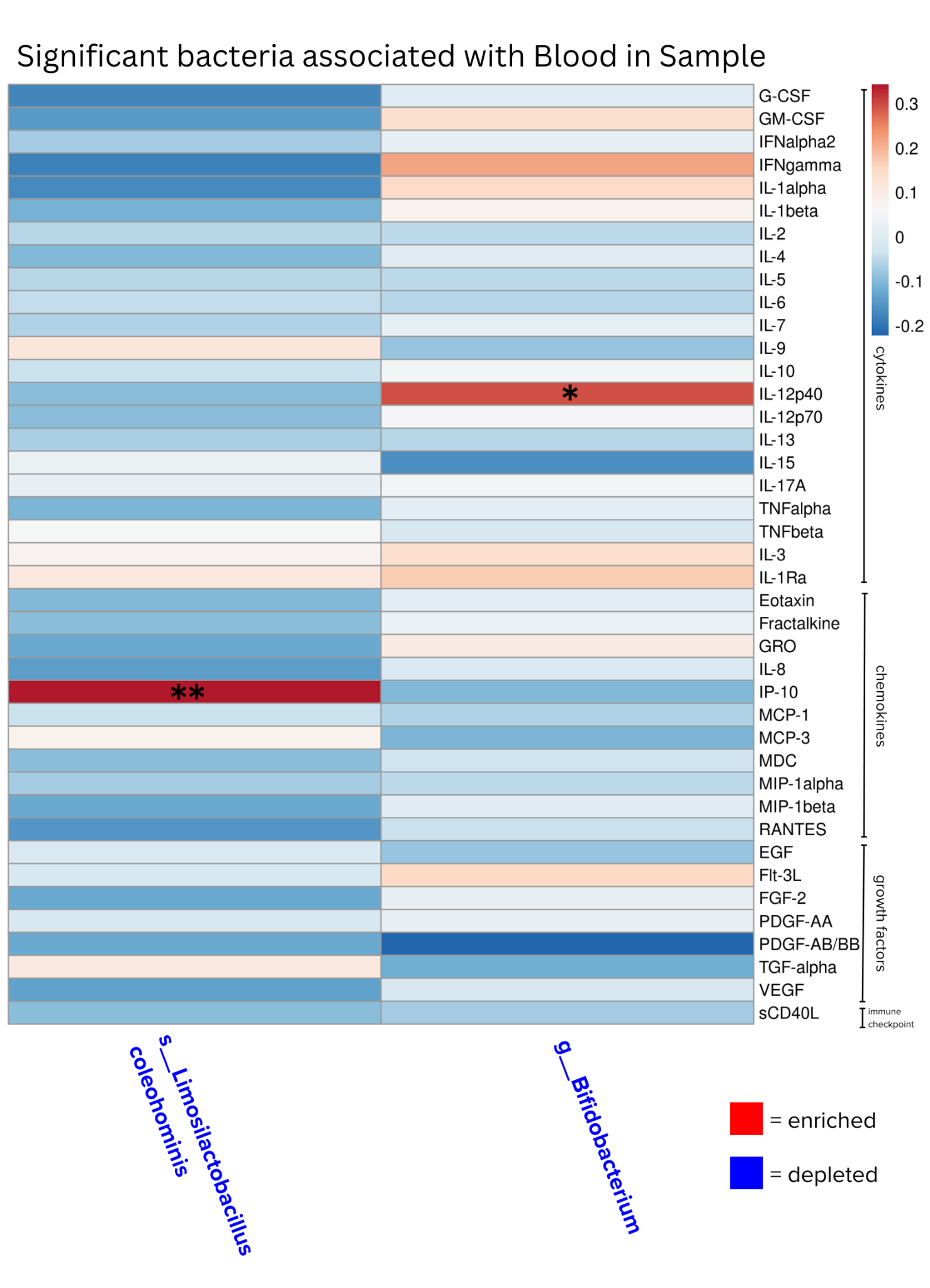


**Figure S28: Heatmap of differentially abundant taxa of blood observed in vaginal samples and correlation to immune markers.** A correlation analysis between cervicovaginal lavage levels of 41 immune proteins with significant differentially abundant vaginal taxa of subjects from analysis groups: **(A)** Blood in the sample. Correlation coefficients (r) were calculated using Spearman’s rank correlation, where positive (red) and negative (blue) correlations are depicted as a heatmap. P-values <0.05 were significant, where * is denoted as <0.05, ** is denoted as <0.01, *** <0.001, and **** <0.0001 p-value. Taxa labels in red signify that a bacterial species was enriched in the samples. Taxa in blue signifies a bacterial species depleted in each analysis group's samples.
